# Chemical Profile, Antibacterial, Antibiofilm, and Antiviral Activities of *Pulicaria crispa* Most Potent Fraction: An In Vitro and In Silico Study

**DOI:** 10.3390/molecules28104184

**Published:** 2023-05-19

**Authors:** Fatma Abo-Elghiet, Areej Rushdi, Mona H. Ibrahim, Sara H. Mahmoud, Mohamed A. Rabeh, Saad Ali Alshehri, Nagwan Galal El Menofy

**Affiliations:** 1 Department of Pharmacognosy and Medicinal Plants, Faculty of Pharmacy (Girls), Al-Azhar University, Cairo 11884, Egypt; 2 Department of Medical Microbiology and Immunology, Faculty of Medicine (Girls), Al-Azhar University, Cairo 11884, Egypt; areejrushdi@azhar.edu.eg; 3 Department of Pharmaceutical Medicinal Chemistry and Drug Design, Faculty of Pharmacy (Girls), Al-Azhar University, Cairo 11884, Egypt; mona.hussein@azhar.edu.eg; 4 Center of Scientific Excellence for Influenza Viruses, Environmental Research and Climate Changes Institute, National Research Center, Giza 12622, Egypt; sarahussein9@yahoo.com; 5 Department of Pharmacognosy, College of Pharmacy, King Khalid University, Abha 62514, Saudi Arabia; mrabeh@kku.edu.sa (M.A.R.); salshhri@kku.edu.sa (S.A.A.); 6 Department of Microbiology and Immunology, Faculty of Pharmacy (Girls), Al-Azhar University, Cairo 11884, Egypt; nagwan.elmenofy@azhar.edu.eg

**Keywords:** *Pulicaria crispa*, antiviral, antibacterial, antibiofilm, GC/MS, influenza A virus

## Abstract

Infectious diseases caused by viruses and bacteria are a major public health concern worldwide, with the emergence of antibiotic resistance, biofilm-forming bacteria, viral epidemics, and the lack of effective antibacterial and antiviral agents exacerbating the problem. In an effort to search for new antimicrobial agents, this study aimed to screen antibacterial and antiviral activity of the total methanol extract and its various fractions of *Pulicaria crispa* (*P. crispa*) aerial parts. The *P. crispa* hexane fraction (HF) was found to have the strongest antibacterial effect against both Gram-positive and Gram-negative bacteria, including biofilm producers. The HF fraction reduced the expression levels of penicillin binding protein (PBP2A) and DNA gyrase B enzymes in *Staphylococcus aureus* and *Pseudomonas aeruginosa*, respectively. Additionally, the HF fraction displayed the most potent antiviral activity, especially against influenza A virus, affecting different stages of the virus lifecycle. Gas chromatography/mass spectrometry (GC/MS) analysis of the HF fraction identified 27 compounds, mainly belonging to the sterol class, with β-sitosterol, phytol, stigmasterol, and lupeol as the most abundant compounds. The in silico study revealed that these compounds were active against influenza A nucleoprotein and polymerase, PBP2A, and DNA gyrase B. Overall, this study provides valuable insights into the chemical composition and mechanism of action of the *P. crispa* HF fraction, which may lead to the development of more effective treatments for bacterial and viral infections.

## 1. Introduction

Infectious diseases have re-emerged as a threat to the world, leading to high morbidity and mortality rates due to epidemics and pandemics [1]. This is mainly due to the crisis of antimicrobial resistance (AMR) and the emergence of new and re-emerging viruses, especially in the absence of preventive vaccines and effective antibiotics and antiviral drugs [1,2]. The inappropriate or excessive use of antibiotics and the lack of new drug discovery have led to the rapid spread of AMR [3]. Antibiotics are crucial in saving lives and allowing modern medicine to exist because chemotherapy, organ transplantation, and surgery would be impossible without them [4]. Biofilms, characterized by self-produced polymeric matrices by bacteria, are strongly associated with antibiotic resistance as they can promote the transmission of antibiotic resistance genes and increase bacterial tolerance to antibiotics [5]. Both Gram-positive and Gram-negative bacteria can produce biofilms, but the most common biofilm-forming bacteria are *Pseudomonas aeruginosa*, *Staphylococcus epidermidis*, and *Staphylococcus aureus* [6,7]. It is important to note that over 65% of all microbial infections are caused by biofilm-forming bacteria [8]. Similarly, viral infections play a crucial role in human diseases, and recent outbreaks have highlighted the critical need to prevent them for public health protection [9]. Hence, there is a dire need for the discovery of innovative antibacterial and antiviral agents that are highly effective, safe, and inexpensive against serious infectious diseases.

Natural products derived from plants, bacteria, fungi, and animals have been utilized for centuries as a source of drugs or drug lead entities, particularly antimicrobial and antiviral agents. This suggests that a return to nature could help resolve the infectious diseases crisis. Penicillin, cephalosporins, doxorubicin, aminoglycosides, amphenicols, tetracyclines, macrolides, and artemisinin are all discovered in natural products [10]. Moreover, the majority of approved synthetic antiviral drugs, such as acyclovir, ganciclovir, famciclovir, sorivudine, didanosine, zalcitabine, zidovudine, and stavudine, are designed based on natural product parents [11]. In fact, natural product drugs have revolutionized the medical field.

*Pulicaria crispa* (*P. crispa*), a member of the well-known Compositae family, is widely grown in Middle Eastern countries including Egypt, Sudan, and Saudi Arabia. Chemical analysis of the plant has revealed a diverse spectrum of phytoconstituents ranging from lipophilic to hydrophilic and amphoteric substances [12]. *P. crispa* contains different classes of secondary metabolites such as volatile oils, sterols, terpenes, flavonoids, glycosides, tannins, coumarins, and alkaloids, which provide the plant with a wide range of pharmacological effects. Traditionally, *P. crispa* has been used for many years to treat heart disorders, colic, coughs, colds, and excessive sweating by the Sudanese, Egyptians, and Saudis. Furthermore, *P. crispa* is considered a carminative, insect-repellent, and antimicrobial agent [13]. Although *P. crispa* essential oil has shown promising antibacterial activity in recent studies, more research is needed to examine the antimicrobial activity of the total extracts and fractions. Furthermore, there is a lack of research on *P. crispa’s* antiviral properties, despite its traditional use in treating colds. 

Therefore, the main objective of this study was to evaluate the antimicrobial and antiviral activity of the total methanol extract and its different polarity fractions obtained from *P. crispa* aerial parts. Furthermore, the mechanism of action of the most potent fraction was investigated, and its antibiofilm effects and chemical composition were assessed. To supplement these findings, an in silico study was conducted to provide insight into the potential mechanisms of action of the abundant components of the active fraction.

## 2. Results

### 2.1. Antibacterial Activity

#### 2.1.1. Preliminary Antibacterial Screening of *P. crispa* Crude Extract and Fractions

Table 1 showed that the crude methanol extract (CME) of *P. crispa* and its different polarity fractions, including hexane (HF), dichloromethane (DCF), ethyl acetate (EAF), and water (WF), displayed good antibacterial activity against the four bacterial strains tested, as evidenced by their minimum inhibitory concentrations (MIC). Among these fractions, the HF fraction exhibited the highest antibacterial activity, particularly against *Pseudomonas aeruginosa* and *Staphylococcus aureus*, compared to the other fractions. As a result, further investigations using the MIC method will be conducted to evaluate the efficacy of the HF fraction against additional Gram-positive and Gram-negative bacteria. 

#### 2.1.2. Evaluation of Antibacterial Activity of *P. crispa* Most Potent Fraction (HF)

Table 2 indicated that *P. crispa* HF fraction exhibited potent antibacterial activity against all tested bacterial isolates, with the lowest MIC values observed against *Acinetobacter baumannii* (ATCC 19606), *Pseudomonas aeruginosa* (Clinical isolate), and *Staphylococcus aureus* (ATCC 25923). Additionally, MBC (minimum bactericidal concentration) value was found to be twice the MIC value for each bacterial strain, demonstrating the HF fraction’s ability to not only inhibit bacterial growth but also kill the bacteria. Moreover, the HF fraction was found to be more potent than the standard antibiotic amoxicillin against several bacterial strains, including *Acinetobacter baumannii* (ATCC 19606), *Pseudomonas aeruginosa* (clinical isolate), *Proteus mirabilis*, and MRSA (ATCC 43300).

#### 2.1.3. Gene Expression Analysis

**Figure 1:**
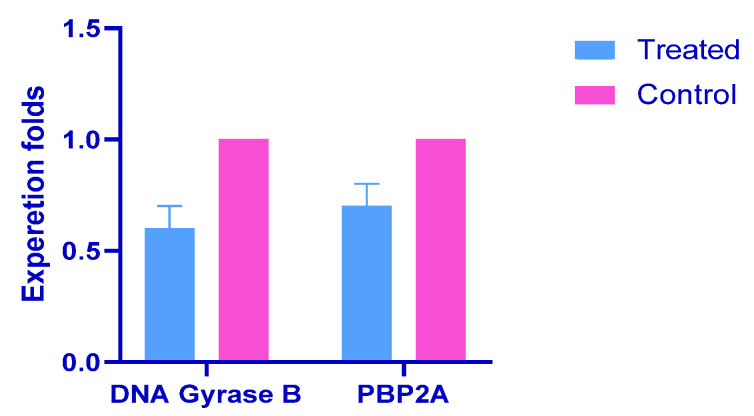
DNA gyrase B and PBP2A relative gene expression levels before and after treatment using *P. crispa* HF fraction.

Real Time-quantitative Polymerase Chain Reaction (RT-qPCR) analysis was employed to investigate the impact of *P. crispa* HF fraction on the expression levels of DNA gyrase B in *P. aeruginosa* and PBP2A in *S. aureus*. The analysis was performed before and after treatment to determine the mechanism of action of the fraction. The findings, illustrated in Figure 1, demonstrated a significant reduction in the expression levels of DNA gyrase B in *P. aeruginosa* and PBP2A in *S. aureus* after treatment with the HF fraction. The RT-qPCR data analysis for DNA gyrase B and PBP2A genes is presented in Tables S1 and S2, respectively.

### 2.2. Antibiofilm Activity

#### 2.2.1. Biofilm Forming Ability

To measure and compare the biofilm formation capability of four bacterial isolates, we employed a standard microtiter plate assay. The biofilm was grown, and the resulting biofilm was evaluated using a colorimetric assay. All the isolates were found to be biofilm producers and were grouped based on their OD value, with *S. aureus* (ATCC 25923) and *Proteus mirabilis* being moderate biofilm producers (2 × ODc < OD ≤ 4 × ODc), while *Acinetobacter baumannii* (ATCC 19606) and *P. aeruginosa* clinical isolate were strong biofilm producers (4 × ODc < OD).

#### 2.2.2. Biofilm Inhibition Activity of *P. crispa* HF Fraction

**Figure 2:**
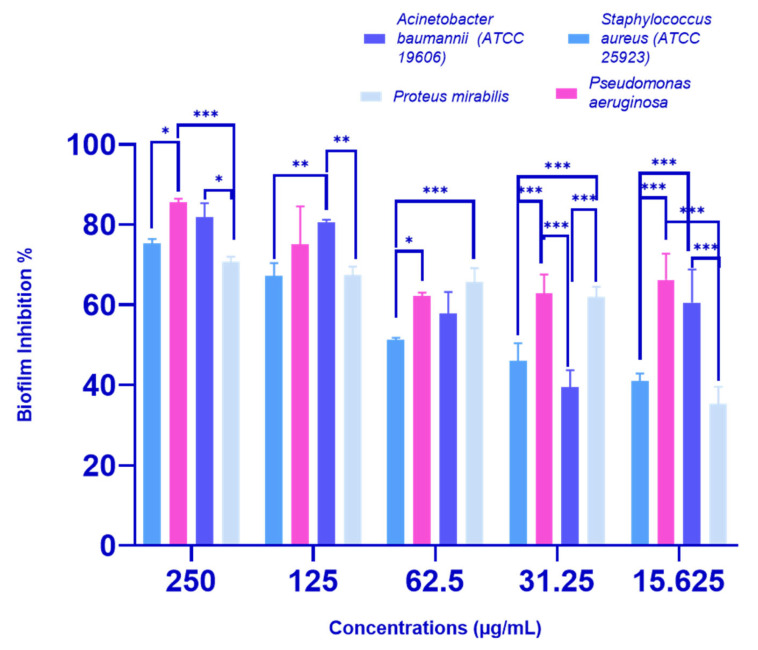
Antibiofilm inhibition effects of different concentrations (ranging from 250 to 15.625 µg/mL) of *P. crispa* HF fraction on biofilm-producing bacteria. The results are presented as the mean biofilm inhibition percentage ± standard deviation (SD) based on three independent experiments (*n* = 3). The figure indicates that there is a significant difference between the effect of the tested concentrations of the HF fraction, as determined by a two-way ANOVA statistical analysis (* *p* < 0.05, ** *p* < 0.005, *** *p* < 0.0005).

The *P. crispa* HF fraction showed a dose-dependent inhibition of biofilm formation in all four tested bacterial strains, as illustrated in Figure 2. At a concentration of 250 µg/mL, HF significantly reduced the percentage of biofilm formation for all strains: *P. aeruginosa* by 85.52%, *A. baumannii* (ATCC 19606) by 81.85%, *S. aureus* (ATCC 25923) by 75.21%, and *P. mirabilis* by 63.33%. Moreover, even at sub-MIC concentrations (31.25 µg/mL for *A. baumannii* and *S. aureus*, and 62.5 µg/mL for *P. aeruginosa* and *P. mirabilis*), the HF fraction still showed significant biofilm inhibition activity, with inhibition percentages ranging from 39.40 to 65.50%, as shown in Figure 2.

#### 2.2.3. Biofilm Detachment Activity of *P. crispa* HF Fraction

**Figure 3:**
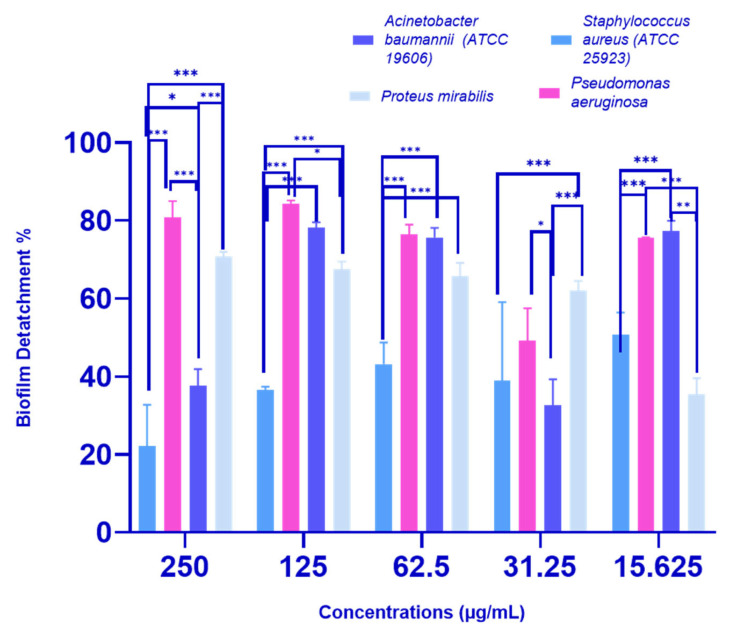
Antibiofilm detachment effects of different concentrations (ranging from 250 to 15.625 µg/mL) of *P. crispa* HF fraction on biofilm-producing bacteria. The results are presented as the mean biofilm detachment percentage ± standard deviation (SD) based on three independent experiments (*n* = 3). The figure indicates that there is a significant difference between the effect of the tested concentrations of the HF fraction, as determined by a two-way ANOVA statistical analysis (* *p* < 0.05, ** *p* < 0.005, *** *p* < 0.0005).

The *P. crispa* HF fraction was effective in inducing biofilm detachment in all tested bacterial strains, as shown in Figure 3. The highest percentage of biofilm detachment was observed at a concentration of 250 µg/mL, with percentages of 22.12%, 37.57%, 70.75%, and 80.84% recorded for *S. aureus* (ATCC 25923), *A. baumannii* (ATCC 19606), *Proteus mirabilis*, and *P. aeruginosa* clinical isolates, respectively. At sub-MIC concentrations (31.25 µg/mL for *A. baumannii* (ATCC 19606) and *S. aureus* (ATCC 25923), and 62.5 µg/mL for *P. aeruginosa* and *P. mirabilis*), the biofilm detachment percentages were 38.87%, 32.68%, 65.83%, and 76.38%, respectively. Significant differences were observed in biofilm detachment activity against all tested strains at nearly all concentrations.

### 2.3. Antiviral Activity

#### 2.3.1. Cytotoxicity Activity

**Figure 4:**
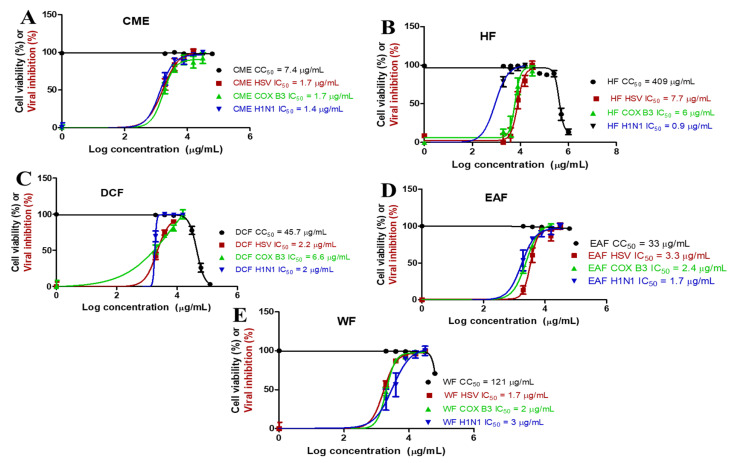
Half maximal cytotoxic concentration (CC_50_) on MDCK cell lines and half maximal inhibitory concentration (IC_50_) against HSV-1, COX B3, and Influenza A virus (H1N1) of the tested *P. crispa* (**A**) crude methanol extract, (**B**) hexane fraction, (**C**) dichloromethane fraction, (**D**) ethyl acetate fraction, and (**E**) water fraction. Both CC_50_ and IC_50_ values were calculated by plotting log concentrations of the tested extract/fractions vs. normalized response (variable slope) using nonlinear regression analysis through GraphPad Prism software (version 5.01).

The cytotoxicity assay was performed on Madin–Darby canine kidney (MDCK) cell lines, and the CC_50_ values were determined for each extract and fraction. The CC_50_ represents the concentration of the extract or fraction required to cause a 50% reduction in cell viability. As illustrated in Figure 4, the CME extract showed the highest cytotoxicity with a CC_50_ of 7.4 µg/mL. Conversely, the HF fraction exhibited the lowest cytotoxicity with a CC_50_ of 409 µg/mL.

#### 2.3.2. Antiviral Screening (Determination of 50% Inhibitory Concentration, IC_50_)

The antiviral activity of *P. crispa* extract and fractions was evaluated through a screening process against several viruses, including Herpes simplex virus type 1 (HSV-1), Coxsackie B virus type 3 (COX B3), and Influenza A virus (H1N1). For each virus, the IC_50_ value, representing the concentration of the extract or fraction required to inhibit viral replication by 50%, was determined. The results, as shown in Figure 4, revealed promising inhibitory activity against all tested viruses, with IC_50_ values ranging from 0.9 to 7.7 µg/mL. However, the extract and fractions showed greater effectiveness against the Influenza A virus, based on the lower IC_50_ values.

Furthermore, the selective index (SI) of *P. crispa* extract and fractions against influenza A virus was calculated as presented in Table 3. SI is the ratio of the CC_50_ to the IC_50_. Based on the obtained IC_50_ and SI values, the *P. crispa* HF fraction showed the most effectiveness and selectivity against the Influenza A virus.

#### 2.3.3. Time of Addition (TOA) Effect of *P. crispa* HF Fraction on Influenza A Virus

**Figure 5:**
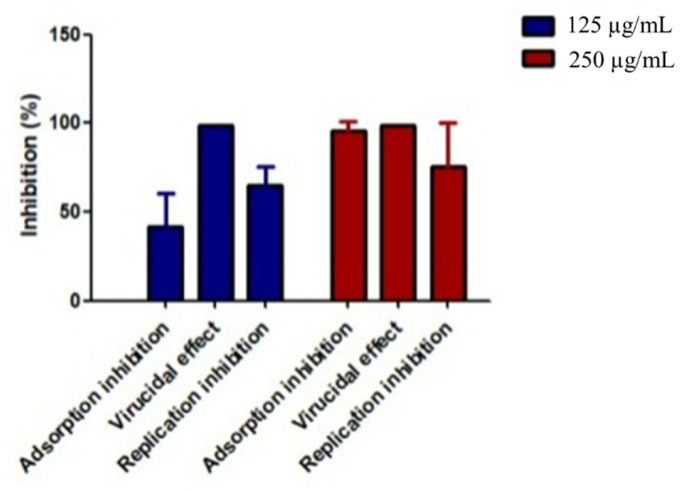
The time of addition (TOA) effect of *P. crispa* HF fraction on the infection cycle of influenza A virus (H1N1).

Due to the high efficacy demonstrated by *P. crispa* extract and fractions against the Influenza A virus, particularly the HF fraction with the greatest potency and selectivity (based on IC_50_ and SI values), further investigation into its anti-Influenza A mechanism was carried out. This involved examining the effects of pretreatment, post-infection, and competition protocols, using two doses of the HF fraction (250 and 125 µg/mL) in the TOA assay. The results showed that the HF fraction inhibited virus entry and replication and also exhibited virucidal activity (the ability of the antiviral agent to directly inactivate the virus), as illustrated in Figure 5. At a concentration of 125 µg/mL, the order of antiviral activity was virucidal activity > replication inhibition > adsorption inhibition, while at a concentration of 250 µg/mL, the order was virucidal activity > adsorption inhibition > replication inhibition.

### 2.4. Chemical Profiling of P. crispa HF Fraction Using GC/MS Analysis

**Figure 6:**
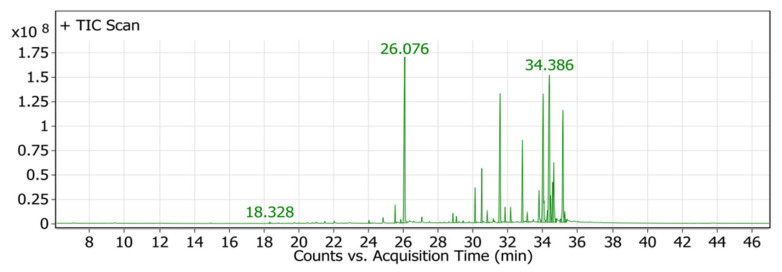
GC/MS chromatogram of *P. crispa* HF fraction.

The GC/MS analysis of the *P. crispa* HF fraction has identified 27 different compounds from various classes, including sterols, hydrocarbons, alcohols, and fatty acids. These compounds have been listed in Table 4, and their chromatogram is shown in Figure 6. The most abundant compounds were β-sitosterol (17.89%), phytol (15.65%), stigmasterol (13.13%), and lupeol (12.89%). The identification of these compounds was performed by comparing their mass spectral fragmentation patterns with those of known compounds listed in the Wiley and NIST Mass Spectral Library database. Furthermore, Table 5 presents the structures and biological activities of the main phytoconstituents identified in the HF fraction.

### 2.5. In Silico Study

Depending on the promising anti-influenza A activity of *P. crispa* HF fraction and its ability to downregulate the expression of genes associated with gyrase B and PBP2A, we conducted a molecular docking study to investigate the potential activities of its most abundant phytoconstituents (β-sitosterol, phytol, stigmasterol, and lupeol) against these bacterial and viral target proteins, providing potential leads for experimental testing.

#### 2.5.1. Molecular Docking against DNA Gyrase B

The most abundant compounds of HF fraction were docked into the ATP binding sites of the *P. aeruginosa* DNA gyrase B protein, with docking scores ranging from −10.84 to −12.38 kcal/mol (Table S3). Arginine residues at positions 78 and 138 are important for enzymatic activity [11]. All the tested compounds, including the inhibitor ligand EZ6, bound to either Arg78 or Arg138, except for lupeol. Lupeol formed two conventional hydrogen bonds with Asn48 and Val45 and nine alkyl bonds with Ile96, Val169, Val45, Val73, Ile80, and Pro8 (Table S3, Figure S1).

**Figure 7:**
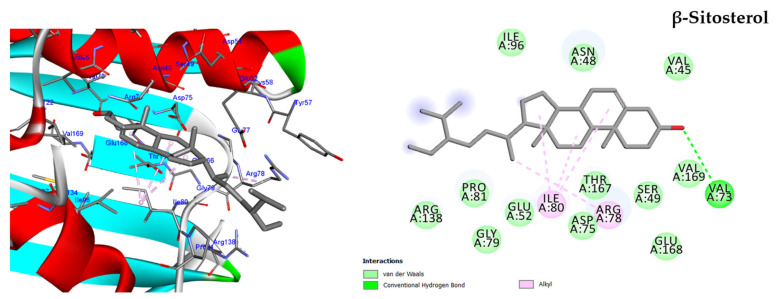
Two-dimensional and three-dimensional images of β-sitosterol docked into the active sites of DNA gyrase B enzyme.

Interestingly, β-Sitosterol achieved the highest docking score of −12.38 kcal/mol, which is nearly equal to the score of inhibitor ligand EZ6 (−12.40 kcal/mol). The hydroxyl group of β-sitosterol formed a conventional hydrogen bond with Val73, and the two rings of the phenanthrene moiety formed two alkyl interactions with Ile80. Additionally, the methyl group at position 13 bound through hydrophobic interaction with the essential amino acid Arg78 (Figure 7).

#### 2.5.2. Molecular Docking against Penicillin-Binding Protein (PBP2A)

**Figure 8:**
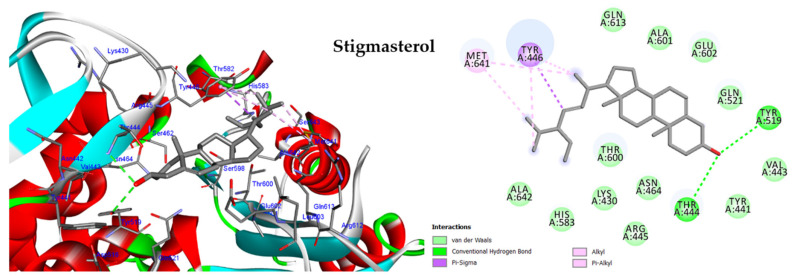
Two-dimensional and three-dimensional images of stigmasterol docked into the active sites of the PBP2A enzyme.

Penicillin-binding protein (PBP2A), one of the transpeptidases, has an active site groove consisting of three important amino acids: Met-641, Thr-600, and Tyr-446 [12]. The molecular docking investigation was conducted on the four tested phytoconstituents against PBP2A using ceftobiprole as an inhibitor ligand. All the tested compounds showed significant binding to the active site groove, with docking scores ranging from −12.03 to −15.65 kcal/mol. Furthermore, all the compounds bound to the crucial amino acid Tyr-446 (Figure 8 and Figure S2, Table S4). Stigmasterol exhibited the lowest binding energy —15.65 kcal/mol compared to other tested compounds and ceftobiprole (Table S4), which formed two hydrogen bonds with Try519 and Thr444 and bound to Tyr446 via three hydrophobic interactions and to Met641 via two alkyl bonds. In contrast, phytol showed the lowest docking score of −12.03 kcal/mol.

#### 2.5.3. Molecular Docking against Influenza A Virus Nucleoprotein (NP)

**Figure 9:**
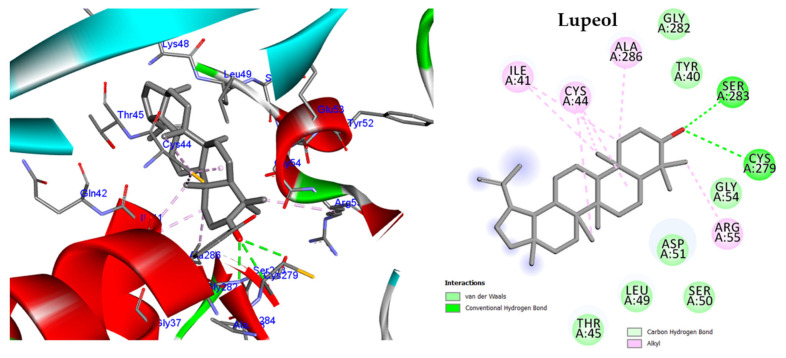
Two-dimensional and three-dimensional images of lupeol docked into the active sites of Influenza A virus nucleoprotein (NP).

To investigate how β-Sitosterol, phytol, stigmasterol, and lupeol bind to the active site of influenza A virus NP, we conducted docking experiments. The results indicated that all four molecules interact with the NP active pocket, exhibiting binding energies ranging from −8.32 to −9.08 Kcal/mol. Notably, these binding energies are lower than that of inhibitor ligand (B7O), which exhibited a binding energy of −6.31 Kcal/mol (Table S5). Interestingly, all four compounds bound to the I41-binding pocket of the influenza A virus NP, which is comprised of Ile41, Asp51, Gly54, Arg55, Ser283, Val285, Ala286, and Cys44 residues (Figure S3). Furthermore, each molecule formed a hydrogen bond with Ser283 and at least one alkyl bond with Cys44. Among the four compounds, lupeol exhibited the highest docking score of −9.08 kcal/mol and formed three hydrogen bonds with Ser283 and Cys279, as well as seven alkyl interactions with Cys44, Ile41, and Arg55 (Figure 9).

#### 2.5.4. Molecular Docking against Influenza A Virus Polymerase

**Figure 10:**
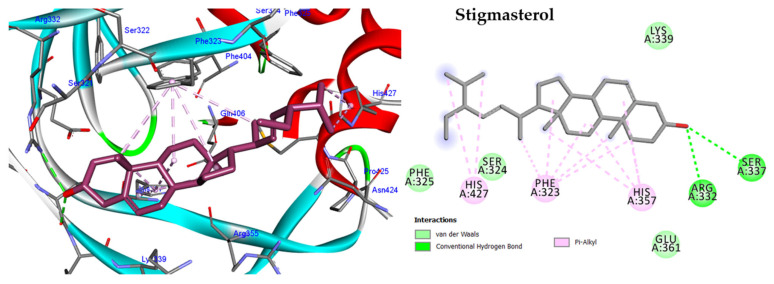
Two-dimensional and three-dimensional images of stigmasterol docked into the active sites of Influenza A virus polymerase.

The influenza A virus relies on a viral polymerase enzyme to replicate and transcribe its RNA genome inside infected cells. This enzyme consists of three subunits, namely PB1, PB2, and PA, which work together to carry out these crucial processes within the cell nucleus [13]. Our docking study focused on the PB2 subunit, which contains the cap-binding domain for 7-methyl GTP (m7 GTP) at the 5′ end of the host pre-mRNA, and it comprises several key residues, including His357 and Phe323 [14]. The docking results showed that β-sitosterol, phytol, stigmasterol, and lupeol exhibited interactions with the PB2 subunit similar to those of the viral replication inhibitor 21G. Moreover, these compounds exhibited higher docking scores (−9.68, −10.68, −8.38, and −11.04 Kcal/mol, respectively) than the inhibitor 21G (−8.07 Kcal/mol) and formed hydrophobic interactions with His357 and Phe323 (Figure S4 and Table S6). Notably, stigmasterol showed the most favourable binding energy among the tested compounds and formed multiple hydrogen bonds and Pi-alkyl bonds with the PB2 subunit residues (Figure 10). Conversely, lupeol had the least favourable binding energy.

## 3. Discussion

Bacterial and viral infections are still a significant threat to global health, with antibiotic resistance and a lack of effective antiviral treatments exacerbating the issue [15]. Medicinal plants have emerged as a promising source for discovering new drugs, and *P. crispa* is one such plant that has been traditionally used in the Middle East for its various health benefits [16]. Our study aimed to investigate the antibacterial and antiviral properties of *P. crispa* crude extract and fractions, identify the most effective fraction, and explore its chemical profile and mechanism of action.

In this study, the potential antibacterial activity of crude extract (CME) and various fractions (HF, DCF, EAF, and WF) derived from *P. crispa* was investigated against both Gram-positive and Gram-negative bacteria. The findings revealed that the HF fraction had the strongest antibacterial effect, especially, against *S. aureus* (clinical isolate) and *P. aeruginosa* (ATCC 29853), with MIC of 62.5 µg/mL for both bacteria. This activity was even stronger than the reference standard used, amoxicillin, which had an MIC of >500 and 125 µg/mL, respectively. These results were consistent with recently published studies that reported the good antimicrobial properties of *P. crispa* against bacteria and fungi [17]. The HF fraction was further tested against additional Gram-positive and Gram-negative bacteria and demonstrated promising antibacterial activity, with MIC values ranging from 15.6 to 125 µg/mL, particularly against *P. aeruginosa* (Clinical isolate), which had the lowest MIC of 15.6 µg/mL. Additionally, the minimum bactericidal concentrations (MBC) were determined and were found to be twice the MIC values for each bacterial strain. This indicated the ability of HF fraction not only to inhibit bacterial growth but also to kill the bacteria. The MBC/MIC ratio, which is a valuable parameter in evaluating the effectiveness of antimicrobial agents, was calculated for the HF fraction against all bacteria tested. The results showed that the HF fraction exhibited a bactericidal effect against all tested bacteria, highlighting its potential as a potent antimicrobial agent. A ratio of 1:1 or 2:1 is typically associated with strong bactericidal activity, while a ratio of 4:1 or higher indicates bacteriostatic activity [18].

To confirm the bactericidal activity of the HF fraction, this study investigated the expression levels of PBP2A and gyrase B in *S. aureus* and *P. aeruginosa*, respectively, using the RT-qPCR technique. PBP2A is a transpeptidase enzyme responsible for cell wall biosynthesis in bacterial species such as *S. aureus.* Inhibition of PBP2A prevents proper formation of the cell wall, leading to cell lysis and death [19]. Gyrase B is a topoisomerase enzyme involved in DNA replication, transcription, and repair, and is a target for antibiotics against *P. aeruginosa* [20]. After treatment with the HF fraction at its MIC concentration, there was a significant reduction in the expression levels of PBP2A and gyrase B in *S. aureus* and *P. aeruginosa*, respectively, indicating the bactericidal activity of the HF fraction.

The chemical profile of *P. crispa* HF fraction was investigated using the GC/MS technique, and the results revealed the identification of 27 compounds, mainly belonging to the sterol class. The most abundant compounds identified were β-sitosterol, phytol, stigmasterol, and lupeol. These compounds are known to have antibacterial and antiviral properties, as reported in Table 5, which could explain the observed effects of the HF fraction. Interestingly, the results of the GC/MS analysis were consistent with previous research on *P. crispa*, which also reported high levels of sterols and/or triterpenes in it [21]. In addition, studies have reported that the Compositae family commonly contains sitosterol and stigmasterol [22]. 

The anti-PBP2A and anti-DNA gyrase B effects of the HF fraction could be attributed to its main phytoconstituents, including β-sitosterol, phytol, stigmasterol, and lupeol. In order to investigate the effects of these compounds on PBP2A and DNA gyrase B, a molecular docking study was conducted, and the results indicated that all tested compounds had a strong affinity for the target proteins. The docking scores ranged from −10.84 to −12.38 kcal/mol against DNA Gyrase B, and −12.03 to −15.65 kcal/mol against PBP2A. The highest docking score was observed for β-sitosterol against DNA Gyrase B and Stigmasterol against PBP2A. Interestingly, the docking score of Stigmasterol against PBP2A was higher than that of the ceftobiprole ligand, and β-sitosterol was nearly equal to the co-crystal ligand EZ6. These findings suggest that the main phytoconstituents of the HF fraction could be promising candidates for developing antibacterial agents, particularly, against PBP2A and DNA gyrase B enzymes.

Biofilms are communities of microorganisms that can cause persistent infections on surfaces and are highly resistant to conventional antibiotics [23]. To find an alternative treatment, the antibiofilm activity of *P. crispa* HF fraction was evaluated against four biofilm producer bacteria, including two moderate biofilm producers (*S. aureus* (ATCC 25923) and *Proteus mirabilis*) and two strong biofilm producers (*Acinetobacter baumannii* (ATCC 19606) and *P. aeruginosa*), and was found to significantly inhibit and detach biofilms in a dose-dependent manner. At a concentration of 250 µg/mL, the HF fraction significantly inhibited biofilm of all tested strains, with percentages ranging from 63.33 to 85.52%. Even at sub-MIC concentrations, the HF fraction still displayed significant biofilm inhibition activity, ranging from 39.40 to 65.50%. The HF fraction also exhibited biofilm detachment activity in all four bacterial strains, with detachment percentages ranging from 22.12 to 80.84% at a concentration of 250 µg/mL, while sub-MIC concentrations showed detachment percentages of 38.87 to 76.38%. Based on the inhibition and detachment percentages, *P. crispa* HF fraction demonstrated promising antibiofilm activity, with values above 50% considered good activity [24]. These results suggest that the *P. crispa* HF fraction could be a natural alternative to conventional antibiotics for managing bacterial infections associated with biofilm formation.

Furthermore, the cytotoxicity and the potential antiviral activity of *P. crispa* crude extract (CME) and various fractions (HF, DCF, EAF, and WF) were investigated. Determining cytotoxicity is crucial when assessing the antiviral potential, as it ensures the safety of potential antiviral agents towards host cells and provides a baseline for selecting doses for further evaluation. The cytotoxicity results (CC_50_) of the tested agents against MDCK cell lines ranged from 7.4 to 409 µg/mL, with the HF fraction having the highest value, indicating the best result in terms of safety towards host cells. Additionally, all tested agents demonstrated antiviral activity against HSV-1, COX B3, and influenza A virus, with the best performance against influenza A virus, as indicated by lower IC_50_ values, suggesting they may be more suitable for treating influenza infections. To determine the most effective and selective agent against the influenza A virus, the selectivity index (SI) was calculated. The SI is the ratio of the CC_50_ to the IC_50_, and higher values indicate greater selectivity and effectiveness against the virus. The calculated SI values for the extract and fractions ranged from 5.3 to 454.4 (Table 3). Based on the obtained IC_50_ and SI values, the *P. crispa* HF fraction showed the most effectiveness and selectivity against the Influenza A virus.

The mechanism of action of *P. crispa* HF fraction against influenza A virus was studied to gain insight into its potential as an antiviral agent. To accomplish this, the TOA effect of HF on the infection cycle of the influenza A virus was analyzed using three different protocols: pre-treatment, post-infection, and competition. The results indicated that HF fraction targeted different stages of the viral replication cycle in its two tested doses, 125 and 250 µg/mL. However, the main mechanism was its virucidal effect. The findings suggest that *P. crispa* HF fraction may be a promising candidate for the development of new antiviral agents against the influenza A virus. 

In addition, a docking study was performed on the most abundant phytoconstituents of the HF fraction, including β-sitosterol, phytol, stigmasterol, and lupeol, against two important proteins in the influenza virus lifecycle, including influenza A virus nucleoprotein (NP) and polymerase. NP plays a crucial role in viral replication and packaging of the RNA genome, while polymerase is responsible for viral transcription and replication [25]. The docking results revealed that all the tested compounds exhibited favourable binding energies, indicating strong binding affinity to the active pockets of NP and polymerase subunits. Interestingly, all four compounds showed similar interactions with the NP active pocket, forming hydrogen bonds with Ser283 and alkyl bonds with Cys44, and binding to the I41-binding pocket of the NP. They also showed docking scores higher than the reference inhibitor B7O, with lupeol exhibiting the highest docking score and forming the most hydrogen bonds and alkyl interactions. These results suggest that lupeol could be a promising inhibitor of the NP protein of the influenza A virus. Similarly, the docking results for the polymerase PB2 subunit showed that all compounds exhibited higher docking scores than the reference inhibitor 21G. The compounds also interacted with key residues within the cap-binding domain of the PB2 subunit, including His357 and Phe323, suggesting that they could interfere with the viral replication and transcription process. In particular, stigmasterol exhibited the lowest binding energy. These results suggest that *P. crispa* HF fraction phytoconstituents could potentially serve as inhibitors of the influenza A virus, with lupeol and stigmasterol showing the most promising results. It is noteworthy that these results provide scientific evidence that supports the traditional use of *P. crispa* for colds [17]. 

One potential limitation of our study is that we did not evaluate the individual or combined activities of the most abundant compounds that comprise the *P. crispa* HF fraction (β-sitosterol, phytol, stigmasterol, and lupeol). As such, we cannot determine whether the observed activity was due to a particular component, or a synergistic effect of all components present in the extract. Future investigations could address this limitation by conducting separate experiments on each of the compounds and testing them in combination.

## 4. Material and Methods

### 4.1. Plant Material

To comply with national and international regulations, the aerial parts of *Pulicaria crispa* were gathered from Saint Catherine, South Sinai, Egypt in March 2021. The plant’s authenticity was confirmed by a plant taxonomist, Mrs. Terase Labib, at Orman Garden in Giza, Egypt. A voucher specimen labelled as (Pc21) was deposited at the herbarium of Pharmacognosy and Medicinal Plants Department, Faculty of Pharmacy (Girls), Al-Azhar University, Cairo, Egypt, for future reference.

### 4.2. Extraction and Fractionation

*P. crispa* aerial parts were shade-dried and crushed into a coarse powder. Then, 300 g of plant material was macerated in methanol until exhaustion. The methanol extract was filtered using Whatman no. 1 filter paper and dried under vacuum using a rotary evaporator. Subsequently, the crude methanol extract (CME, 75 g) was subjected to liquid-liquid partitioning using solvents of increasing polarity, including *n*-hexane, dichloromethane, and ethyl acetate, leading to the isolation of four different fractions: hexane (HF, 3 g), dichloromethane (DCF, 10 g), ethyl acetate (EAF, 13 g), and water (WF, 40 g) [26].

### 4.3. Antibacterial Activity

The antibacterial activity of *P. crispa* crude extract (CME) and its fractions (HF, DCF, EAF, and WF) was primarily screened against several bacterial isolates, including Gram-positive clinical isolate; *Staphylococcus aureus* and Gram-negative bacterial isolates; *Klebsiella pneumonia* (ATCC 700603), *Pseudomonas aeruginosa* (ATCC 29853), and *Escherichia coli* (clinical isolate) to determine their minimum inhibitory concentrations.

The most effective fraction was then tested against additional bacterial isolates, including *Escherichia coli* (ATCC 25922), *Acinetobacter baumannii* (ATCC 19606), Methicillin-resistant *Staphylococcus aureus* (MRSA ATCC 43300), *Staphylococcus aureus* (ATCC 25923), *Pseudomonas aeruginosa* (a clinical isolate), and two biofilm producers clinical isolates (*Pseudomonas aeruginosa* and *Proteus mirabilis*) to determine its minimum inhibitory concentration and minimum bactericidal concentration according to the CLSI reference standards [27].

#### 4.3.1. Determination of Minimum Inhibitory Concentration (MIC)

To determine the MIC of both crude extract and fractions of *P. crispa*, the microbroth dilution assay was used. Initially, 96 multi-well microtiter plates were filled with 100 µL of Muller–Hinton broth (MHB) (Oxoid^®^ Limited, Basingstoke, UK) in each well. Subsequently, the crude extract or fractions dissolved in DMSO (100 µL) were added to the first row of the microtiter plate, and serial dilutions were performed from the first to the twelfth well. Fresh bacterial suspensions (1.5 × 10^8^ CFU/mL, 7 µL per well) were then inoculated into each well. For each bacterial strain, negative and positive controls were conducted, and amoxicillin was used as a reference standard antibiotic at a concentration of 1000 µg/mL. The plates were then incubated at 37 °C for 18 to 24 h, and the MIC was defined as the lowest concentration of the extract or fraction that inhibited bacterial growth. The experiment was repeated twice to ensure the accuracy and reproducibility of the results.

#### 4.3.2. Determination of Minimum Bactericidal Concentrations (MBC)

To determine the minimum bactericidal concentration (MBC) of the most potent fraction, 10 µL of the contents from each well that exhibited no visible growth on multi-well microtiter plates were transferred onto the Muller–Hinton agar (MHA). Subsequently, they were incubated for 24 h at 37 °C. The MBC was determined as the minimum concentration of the fraction at which no bacterial growth was observed on the surface of the agar. To ensure accuracy, the experiments were conducted twice. 

#### 4.3.3. Real Time-Polymerase Chain Reaction (RT-qPCR) Analysis

This experiment evaluated the expression levels of penicillin-binding protein PBP 2A in *Staphylococcus aureus* (ATCC 25923) and gyrase B in *Pseudomonas aeruginosa* (ATCC 29853) using RT-qPCR analysis. 

Bacteria were grown following the protocol of MIC determination in the presence and absence of *P. crispa* HF fraction. Total RNA was extracted from the harvested bacteria using TRIzol reagent (15596018, Invitrogen, Carlsbad, CA, USA) and purified according to the manufacturer’s instructions. The RNA was then reverse transcribed into cDNA using the QuantiTect Reverse Transcription Kit (Qiagen, Germantown, MD, USA) with gene-specific PCR primers for PBP 2A and gyrase B (Table 6). The resulting cDNA and a negative control were subjected to amplification using Maxima SYBR Green/Fluorescein qPCR Master Mix and Rotor-Gene Q (Qiagen, Germantown, MD, USA), and the housekeeping amplicon used was β-actin. The relative gene expression fold change was determined using the ΔΔCt method based on at least three independent RNA samples. The thermal cycling conditions followed the protocol established by Lanoix et al., 2006 [28]. The relative expression levels of the target genes were estimated using the 2^−∆∆ct^ method, and the fold changes were calculated accordingly.

### 4.4. Antibiofilm Activity

To evaluate the antibiofilm activity of *P. crispa* HF fraction, this study employed a biofilm inhibition assay and a biofilm detachment assay.

Initially, all bacterial isolates were screened for their biofilm-forming ability using the crystal violet assay as described by Tawfick et al. [32]. The assay involved growing the bacteria in a flat-bottomed 96-well cell culture microtiter plate containing tryptic soy broth and glucose and staining the resulting biofilm with crystal violet. Then, the following equations were used to categorize the biofilm producers: non-biofilm producers (OD ≤ ODc), weak biofilm producers (ODc < OD ≤ 2 × ODc), moderate biofilm producers (2 × ODc < OD ≤ 4 × ODc), and strong biofilm producers (4 × ODc < OD). Where OD is optical density and ODc is control optical density.

The statistical analysis was conducted using GraphPad Prism 8.0.2 (GraphPad Software, Inc., San Diego, CA, USA).

#### 4.4.1. Biofilm Inhibition Assay

The biofilm inhibition assay was performed as previously reported by Nassima et al. [33]. In brief, the sterile microtiter plates were prepared by adding 100 µL of tryptic soy broth (TSB; Oxoid^®^ Limited, Basingstoke, UK) supplemented with 1% glucose to each well, and then added various concentrations of *P. crispa* HF fraction (ranging from 250 to 15.625 µg/mL) in triplicate. Next, fresh overnight cultures of biofilm-forming bacterial isolates (10^6^ CFU/mL), that had been diluted 1:100 in fresh TSB with glucose, were added to each well. The plates were incubated at 37 °C for 24 h without shaking, and then rinsed three times with phosphate-buffered saline (PBS) to remove loosely attached cells. The remaining biofilm was fixed with absolute methanol and stained with crystal violet. After solubilizing the dye with ethanol, the OD of the resulting solution was determined at 500 nm using a UV spectrophotometer (Optizen pop, Mecasys, Daejeon, South Korea). The percentage of biofilm inhibition was calculated using the following formula:

Biofilm Inhibition % = (OD growth control − OD sample) / OD growth control × 100

#### 4.4.2. Biofilm Detachment Assay

To perform the biofilm detachment assay, A microtiter plate with pre-formed biofilm (grown from fresh overnight cultures of biofilm-forming bacterial isolates diluted 1:100 in TSB with glucose) was incubated for 24 h without shaking. Then, we removed the contents of the wells and added TSB supplemented with 1% glucose and various concentrations of *P. crispa* HF fraction (ranging from 250 to 15.625 µg/mL) in triplicate. After another 24 h incubation, the wells were rinsed with PBS, fixed with methanol, and stained with crystal violet. The OD of the resulting solution was determined at 500 nm using a spectrophotometer. The percentage of biofilm detachment was calculated using the same formula as in the biofilm inhibition assay.

### 4.5. Antiviral Activity

#### 4.5.1. Cell Lines

Vero and Madin–Darby canine kidney (MDCK) cells were maintained in Dulbecco’s modified Eagle’s medium (DMEM; Lonza, Verviers, Belgium) supplemented with 10% Fetal bovine serum (FBS; Gibco, New York, NY, USA) and 1% antibiotic-antimycotic mixture (Lonza, Verviers, Belgium) at 37 °C in a humidified 5% CO_2_ incubator. 

#### 4.5.2. Viruses

Herpes simplex virus type 1 (HSV-1), Coxsackie B virus type 3 (COX B3), and influenza virus A/Puerto Rico/8/34 (H1N1) were kindly provided by the Center of Scientific Excellence for Influenza Virus. To produce virus stocks of HSV-1 and COX B3, Vero cells were cultured in T75 cell culture flasks for 24 h, followed by infection with 0.1 multiplicity of infection of each virus in an infection medium composed of DMEM supplemented with 2% FBS and 1% antibiotic-antimycotic mixture. After 72 h, the supernatant was collected, centrifuged at 2500 rpm, and stored in aliquots for future use.

In the case of the influenza virus, MDCK cells were used instead of Vero cells, and the infection medium was supplemented with 1% trypsin.

#### 4.5.3. Cytotoxicity Assay

The cytotoxicity assay was performed to determine the half maximal cytotoxic concentration (CC_50_) of *P. crispa* crude extract (CME) and fractions (HF, DCF, EAF, and WF) on MDCK cell lines. Briefly, 96-well plates were prepared by seeding each well with 100 µL of cell suspension (3 × 10^5^ cells/mL) and incubating them for 24 h at 37 °C in the presence of 5% CO_2_. The cells were then washed with PBS and treated with serial two-fold dilutions of the fractions in triplicate. After 72 h, the supernatant was discarded, and the cells were fixed with 10% formaldehyde for 1 h at room temperature. The plates were stained with 50 µL of 0.1% crystal violet on a bench rocker, rinsed with water, and dried overnight. The following day, the crystal violet in each well was dissolved with 200 µL methanol, and the absorbance was measured at 570 nm using a multi-well plate reader. Nonlinear regression analysis was performed using GraphPad Prism software (version 5.01) to estimate the CC_50_, or the concentration of *P. crispa* extract/fractions required to reduce cell viability by 50%, by plotting log concentrations of the extract/fractions vs. normalized response (variable slope) [34].

#### 4.5.4. Antiviral Screening Assay (Determination of 50% Inhibitory Concentration, IC_50_)

The assay aimed to determine the 50% inhibitory concentration (IC_50_) values of *P. crispa* extract/fractions against HSV-1, COX B3, and influenza A virus (H1N1). To perform this assay, Vero cells were used for HSV-1 and COX B3 viruses, while MDCK cells were used for the influenza virus. The cells were seeded in 96-well plates and incubated for 24 h before adding serial two-fold dilutions of the tested extract/fractions, along with virus suspensions containing 100 TCID50 of each virus. After 1 h, the mixture was transferred to PBS-rinsed cell monolayers, and the cells were incubated for 72 h. Cell control wells and virus control wells were also included. After 72 h, the cells were fixed with 10% formaldehyde, stained with crystal violet, and the absorbance was measured at 570 nm. The IC_50_ value was calculated by plotting log concentrations of the tested extract/fractions versus normalized response (variable slope) using nonlinear regression analysis through GraphPad Prism software (version 5.01). The IC_50_ value represents the concentration necessary to reduce the viral-induced cytopathic impact by 50% relative to the virus control [34].

The selectivity whichndex (SI),whichch represents the ratio of CC_50_ to IC_50_, was calculated, as it forms an indicator of the safety and efficacy of the tested extract/fractions as antiviral agents.

#### 4.5.5. Time of Addition (TOA) Assay

The TOA assay is a useful technique for determining the stage in the viral replication cycle at which an antiviral agent has an impact. In this experiment, we sought to examine whether the most active *P. crispa* fraction, HF, could disrupt the life cycle of the influenza A virus, which showed the highest selectivity index (SI). To accomplish this goal, we employed three experimental protocols based on the procedures described by Jin et al. [35]: pretreatment, post-infection, and competition methods.

1. For the pretreatment protocol, we incubated MDCK cells with HF fraction at 125 and 250 µg/mL for 2 h at 37 °C before infecting them with the influenza A virus. After removing the supernatant, we added the virus to the cells and allowed it to incubate for 1.5 h. Then, any unabsorbed virus was removed with PBS and replaced with a fresh medium.
2. In the postinfection protocol, we infected MDCK cells with the influenza A virus and added *P. crispa* HF fraction at 125 and 250 µg/mL 1.5 h later.
3. In the competition protocol, we added HF fraction at 125 and 250 µg/mL to the virus during the adsorption period for 1.5 h. Then, any unbound viruses and HF were removed and cultured in the infected MDCK cells in a fresh medium at 37 °C.

After 72 h, we used the crystal violet assay to determine the antiviral efficacy of *P. crispa* HF fraction for all three experimental protocols, as previously explained. 

### 4.6. Gas Chromatography-Mass Spectrometry (GC-MS) Analysis of P. crispa HF Fraction

The GC-MS analysis of the *P. crispa* HF fraction was conducted at the Central Laboratories Network of the National Research Centre in Giza, Egypt, using an Agilent Technologies system that comprised a 7890B gas chromatograph and a 5977A mass spectrometer detector. The GC was fitted with an HP-5MS column measuring 30 m × 0.25 mm with a film thickness of 0.25 μm, and the carrier gas was hydrogen at a rate of 2.0 mL/min. A diluted sample (1% *v*/*v*) was injected in 2 µL under splitless conditions. 

The temperature program involved heating the sample at 50 °C for 5 min, increasing the temperature at a rate of 5 °C/min to 100 °C and holding it for 0 min, then increasing the temperature at a rate of 10 °C/min to 320 °C and holding it for 10 min. The injector and detector temperatures were held at 280 °C and 320 °C, respectively.

The mass spectra were obtained by electron ionization (EI) at 70 eV with a spectral range of *m*/*z* 25–700 and a solvent delay of 6 min. The mass temperature was set at 230 °C, and the Quad temperature was set at 150 °C. The identification of the different constituents was performed by comparing the fragmentation pattern of the spectra with those stored in the Wiley and NIST Mass Spectral Library data.

### 4.7. In Silico Study

Docking analysis was conducted using the Molecular Operating Environment (MOE)-2019.09 software, and the structures of all studied compounds were sketched using the builder keys. The energy of these molecules was reduced using MOE’s default MMFF94x force field, and a conformer search was performed to obtain their 3D conformers. To acquire the codes for each enzyme, the protein data bank was consulted, resulting in the following codes: DNA Gyrase B (PDB ID: 6m1j), PBP2A (PDB ID: 4dki), Influenza A Virus Nucleoprotein (PDB ID: 6j1u), and Influenza PB2 (PDB ID: 4p1u).

The enzymes were then opened in MOE, where water molecules were eliminated, and missing hydrogen atoms were supplied. For virtual screening, MOE’s Docking setup was used, and the standard docking technique was applied. The best positions, based on London dG, were saved, and energy was minimized using MMFF94x, while poses were ranked using the GBVI/WSA dG ranking algorithm. The poses with the highest scores were selected, and protein-ligand interactions in the active site were visualized using Biovia Discovery Studio 2020.

For validation purposes, all enzymes were re-docked with their respective co-crystallized ligands, demonstrating the effectiveness of the docking technique, with DNA Gyrase B having an RMSD of 0.40 Å, PBP2a having an RMSD of 0.86 Å, Influenza A Virus Nucleoprotein having an RMSD of 0.95 Å, and Influenza PB2 having an RMSD of 0.75 Å.

## 5. Conclusions

Bacterial and viral infections remain a significant global health threat, and the search for effective treatments is ongoing. Medicinal plants have emerged as a promising source of new drugs. In this study, the *P. crispa* hexane fraction (HF) demonstrated potent antibacterial activity against both Gram-positive and Gram-negative bacteria, with bactericidal activity observed. The HF fraction reduced the expression levels of PBP2A and DNA gyrase B enzymes, with stigmasterol and β-sitosterol identified as the most effective compounds against them, respectively. It also exhibited antibiofilm activity by inhibiting and detaching biofilms, providing further evidence of its potential as an effective antimicrobial agent. Additionally, the *P. crispa* HF showed promising activity against the influenza A virus, targeting various stages of the virus lifecycle, with a particular ability to induce virucidal activity. These findings highlight the potential of *P. crispa* as a valuable source of novel antimicrobial agents and expand the field of plant-based drug discovery.

## Figures and Tables

**Table 1:** MIC (µg/mL) of *P. crispa* crude extract and fractions against four pathogenic bacterial strains.

Bacterial Strain	MIC (µg/mL)					
	CME	HF	DCF	EAF	WF	Amoxicillin
*Staphylococcus aureus* (Clinical isolate)	125	62.5	62.5	125	125	>500
*Klebsiella pneumonia* (ATCC 700603)	125	125	125	125	125	>500
*Pseudomonas aeruginosa* (ATCC 29853)	125	62.5	125	125	125	125
*Escherichia coli* (Clinical isolate)	125	125	125	125	125	62.5

CME: crude methanol extract; HF: hexane fraction; DCF: dichloromethane fraction; EAF: ethyl acetate fraction; WF: water fraction.

**Table 2:** MIC and MBC (µg/mL) of *P. crispa* HF fraction against seven pathogenic bacterial strains.

Bacterial Strain	HF			Amoxicillin
	MIC (µg/mL)	MBC (µg/mL)	MBC/MIC	MIC (µg/mL)
Gram-negative bacterial isolates				
*Escherichia coli* (ATCC 25922)	125	250	2	62.5
*Acinetobacter baumannii* (ATCC 19606)	62.5	125	2	>500
*Pseudomonas aeruginosa* (Clinical isolate)	15.6	31.25	2	62.5
*Pseudomonas aeruginosa* (Biofilm producer)	125	250	2	62.5
*Proteus mirabilias* (Biofilm producer).	125	250	2	>500
Gram-positive bacterial isolates				
*Staphylococcus aureus* (ATCC 25923)	62.5	125	2	≤7.8125
MRSA (ATCC 43300)	125	250	2	>500

MRSA: methicillin resistant *Staphylococcus aureus.*

**Table 3:** The CC_50_, IC_50_, and SI of *P. crispa* extract and fractions against influenza A virus.

Sample	CC_50_ (µg/mL)	IC_50_ (µg/mL)	SI
CME	7.4	1.4	5.3
HF	409	0.9	454.4
DCF	45.7	2	22.85
EAF	33	1.7	19.4
WF	121	3	40.3

CC_50_: half maximal cytotoxic concentration, IC_50_: half maximal inhibitory concentration, and SI: selectivity index.

**Table 4:** Identification of phytoconstituents of *P. crispa* HF fraction using GC/MS.

Peak No.	Rt (min.)	Molecular Formula	Relative Area (%)	Compound
1	18.328	C_15_H_26_O	0.1	Farnesol
2	24.027	C_16_H_34_O	0.19	1-Hexadecanol
3	25.530	C_20_H_42_O	0.86	Dihydrophytol
4	25.848	C_18_H_38_O	0.29	1-Octadecanol
5	26.076	C_20_H_40_O	15.65	Phytol
6	28.846	C_13_H_18_O_6_	0.48	1-Monoferuloylglycerol
7	29.036	C_22_H_46_O	0.35	Docosanol
8	29.430	C_19_H_38_O_4_	0.11	1-Monopalmitin
9	30.113	C_24_H_50_	2.25	Tetracosane
10	30.500	C_24_H_50_O	3.81	1-Tetracosanol
11	30.811	C_21_H_42_O_4_	0.71	Glycerol monostearate
12	31.555	C_30_H_50_	12.05	Squalene
13	31.836	C_30_H_62_	0.84	Triacontane
14	32.162	C_32_H_66_	0.83	Dotriacontane
15	32.838	C_35_H_72_	5.97	Pentatriacontane
16	33.111	C_29_H_50_O_2_	0.69	α-Tocopherol
17	33.786	C_28_H_48_O	2.7	Campesterol
18	34.021	C_29_H_48_O	13.13	Stigmasterol
19	34.272	C_30_H_50_O	1.00	β-Amyrin
20	34.386	C_29_H_50_O	17.89	β-Sitosterol
21	34.454	C_29_H_48_O	1.41	Isofucosterol
22	34.568	C_30_H_50_O	4.38	α-Amyrin
23	34.773	C_30_H_50_O	0.29	Cycloartenol
24	35.023	C_37_H_76_O	0.25	1-Heptatriacotanol
25	35.160	C_30_H_50_O	12.89	Lupeol
26	35.266	C_21_H_38_O_4_	0.65	1-Monolinolein
27	35.334	C_26_H_44_O_5_	0.23	Ethyl iso-allocholate

**Table 5:** The structures and the biological activities of the major phytoconstituents identified in *P. crispa* HF fraction.

Compound	Biological Activity	Reference
β-Sitosterol 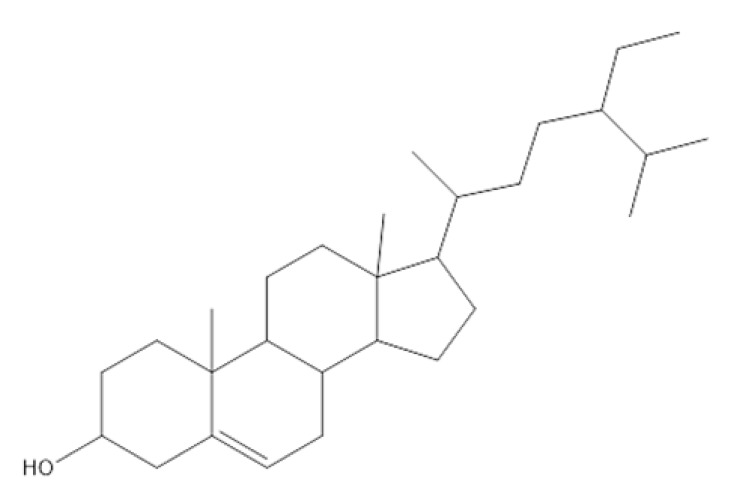	Antibacterial, antifungal, anti-inflammatory, anticancer, antihyperlipidemic, anti-atherosclerosis, and antidiabetic activities.	[1]
Phytol 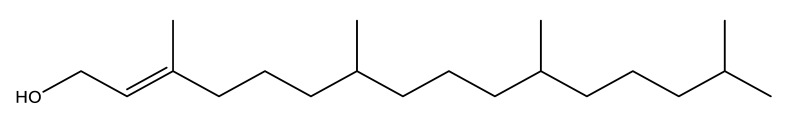	Antibacterial activity via inducing bacterial oxidative cell death. It elevates the level of bacterial intracellular reactive oxygen species (ROS) and transient NADH depletion.	[2]
	Cytotoxic activity on different cancer cell lines in a concentration dependent manner, particularly breast cancer.	[3]
	Antiviral activity against HSV (Herpes Simplex virus).	[4]
	Antioxidant and antifungal activity.	[5]
Stigmasterol 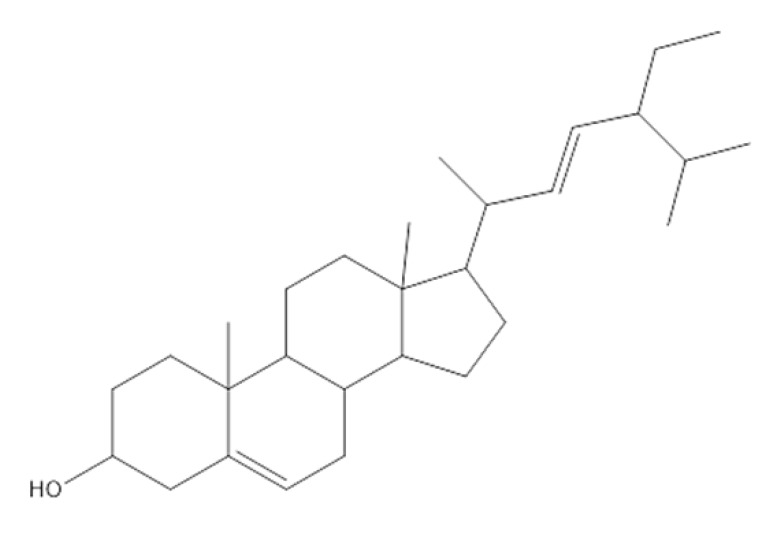	Antibacterial and antifungal activity.	[6]
	Anti-inflammatory activity.	[7]
	Antiviral activity.	[8]
	Antitumor activity.	[9]
Lupeol 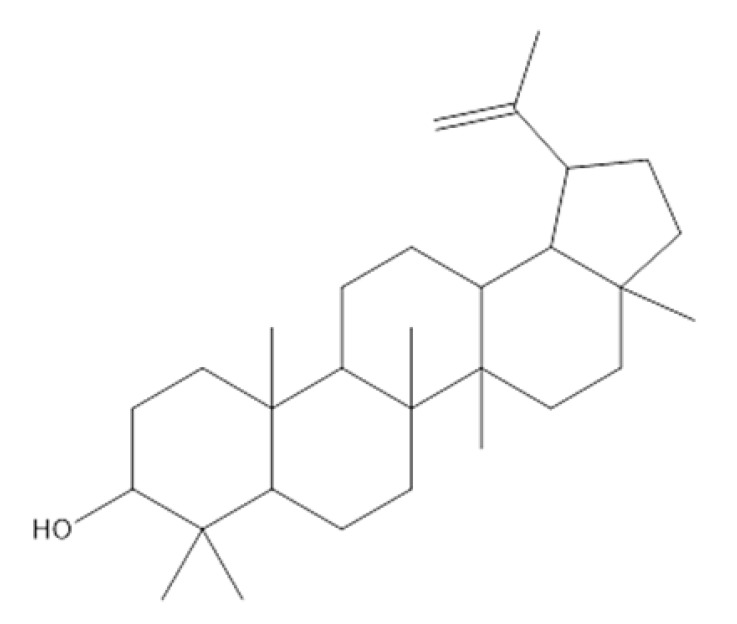	Antimicrobial, antiprotozoal, anti-inflammatory, antitumor, cardioprotective, and hepatoprotective activities.	[10]

**Table 6:** Primers sequences of representative genes used in RT-qPCR analysis.

Gene	Sequence	Reference
β-actin	CCTGCGGCATTCACGAAACTAC	[29]
	ACTCCTGCTTGCTGATCCACAAT	
PBP 2A	CCGCTGATCTTGATTGAATAG	[30]
	ATGCGTTTTCATCCCCTCTG	
DNA-Gyrase B	CAACTTCATCGCCCATTAGG	[31]
	GGGAGAAATGAACCCAGAAC	

## Data Availability

Data are available upon request from the authors.

## Supplementary Materials

The following supporting information can be downloaded at: https://www.mdpi.com/article/10.3390/molecules28104184/s1, Figure S1: Two-dimensional and three-dimensional images of β-sitosterol, phytol, stigmasterol, lupeol, and EZ6 docked into the active sites of DNA gyrase B enzyme, Figure S2: Two-dimensional and three-dimensional images of β-sitosterol, phytol, stigmasterol, lupeol, and ceftobiprole docked into the active sites of PBP2A enzyme, Figure S3: Two-dimensional and three-dimensional images of β-sitosterol, phytol, stigmasterol, lupeol, and B7O docked into the active sites of Influenza A virus nucleoprotein (NP), Figure S4: Two-dimensional and three-dimensional images of β-sitosterol, phytol, stigmasterol, lupeol, and 21G docked into the active sites of Influenza A virus polymerase; Table S1: RT-qPCR data analysis using double delta Ct analysis of DNA gyrase B gene, Table S2: RT-qPCR data analysis using double delta Ct analysis of penicillin-binding proteins (PBP2A) gene, Table S3: Types of interactions and docking energy scores of β-sitosterol, phytol, stigmasterol, lupeol, and EZ6 against DNA gyrase B, Table S4: Types of interactions and docking energy scores of β-sitosterol, phytol, stigmasterol, lupeol, and ceftobiprole against PBP2A, Table S5: Types of interactions and docking energy scores of β-sitosterol, phytol, stigmasterol, lupeol, and B7O against Influenza A virus nucleoprotein (NP), Table S6: Types of interactions and docking energy scores of β-sitosterol, phytol, stigmasterol, lupeol, and 21G against Influenza A virus polymerase.
