# Supplementary material for: Chemical Profile, Antibacterial, Antibiofilm, and Antiviral Activities of Pulicaria crispa Most Potent Fraction: An In Vitro and In Silico Study
Source: Molecules. 2023 May 19;28(10):4184. doi: 10.3390/molecules28104184 (PMC10224259; doi:10.3390/molecules28104184)
Supplement: Supplementary file 1 [file molecules-28-04184-s001.zip › molecules-2361930-supplementary.pdf]

Chemical profile, antibacterial, antibiofilm, and antiviral activities of *Pulicaria crispa* most potent fraction: An in-vitro and in-silico study

Supplementary material

Table S1. RT-qPCR data analysis using double delta Ct analysis of DNA gyrase B gene.

Samples	Gene being Tested Experimental (TE)	Gene being Tested Control (TC)	Housekeeping Gene Experimental (HE)	Housekeeping Gene Control (HC)	ΔCt values for the experimental (ΔCTE)	ΔCt values for the control (ΔCTC)	Delta Ct Value (ΔΔCt)	2^-ΔΔCt (expression fold change)
**Control**	24.8	21.7	24.3	21.2	0.5	0.5	0	1.0
**Treated**	25.3	21.7	24	21.2	1.3	0.5	0.8	0.6

Table S2. RT-qPCR data analysis using double delta Ct analysis of penicillin-binding proteins (PBP2A) gene.

Samples	Gene being Tested Experimental (TE)	Gene being Tested Control (TC)	Housekeeping Gene Experimental (HE)	Housekeeping Gene Control (HC)	ΔCt values for the experimental (ΔCTE)	ΔCt values for the control (ΔCTC)	Delta Ct Value (ΔΔCt)	2^-ΔΔCt (expression fold change)
**Control**	32	22.1	31.4	21.5	0.6	0.6	0	1.0
**Treated**	32.2	22.1	31.1	21.5	1.1	0.6	0.5	0.7

Table S3. Types of interactions and docking energy scores of β-sitosterol, phytol, stigmasterol, lupeol, and EZ6 against DNA gyrase B.

**DNA Gyrase B, PDB ID: 6m1j**					
**Compound**	β-Sitosterol	Phytol	Stigmasterol	Lupeol	Co- crystal Ligand (EZ6)
**Docking energy score (Kcal/ mol)**	-12.38	-10.84	-11.25	-11.25	-12.40
	Val73/H-Bond/3.33	Arg138/H-Bond/3.19	Ile80/Alkyl/4.57<br>Ile80/Alkyl/5.10	Asn48/ H-Bond/3.03	Arg78/Attractive charge/4.70<br>Asp75/H-Bond/3.14

**Amino acids/ Bond type/ Distance (Å)**	Ile80/Alkyl/4.81<br>Ile80/Alkyl/5.00<br>Ile80/Alkyl/5.18<br>Arg78/Alkyl/3.92	Val73/Alkyl/4.12<br>Val169/Alkyl/4.72<br>Ile80/Alkyl/4.08<br>Pro81/Alkyl/4.23	Val169/Alkyl/5.08<br>Arg78/Alkyl/4.66<br>Val73/Alkyl/5.14<br>Val169/Alkyl/5.26<br>Val45/Alkyl/5.40	Val45/H-Bond/2.87<br>Ile80/Alkyl/4.51<br>Ile96/Alkyl/4.62<br>Ile80/Alkyl/4.72<br>Ile80/Alkyl/5.07<br>Val169/Alkyl/5.26<br>Val45/Alkyl/4.86<br>Val73/Alkyl/4.77<br>Ile80/Alkyl/3.78<br>Pro81/Alkyl/4.97	Asp75/H-Bond/2.95<br>Pro81/C-H-Bond/3.45<br>Pro81/C-H-Bond/3.52<br>Gly79/C-H-Bond/3.52<br>Gly79/C-H-Bond/3.44<br>Asn48/Halogen/3.43<br>Arg78/Pi-Cation/3.12<br>Glu52/Pi-Anion/4.52<br>Thr167/ Pi-Donor Hydrogen Bond/3.60<br>Ile80/Pi-Sigma/3.80<br>Pro81/Alkyl/4.77<br>Pro81/Alkyl/4.55<br>Ile96/Akyl/4.54<br>Ile80/Pi-Alkyl/4.71<br>Ile80/Pi-Alkyl/4.92<br>Pro81/Pi-Alkyl/4.80<br>Ile80/Pi-Alkyl/4.74<br>Ile96/Pi-Alkyl/5.15

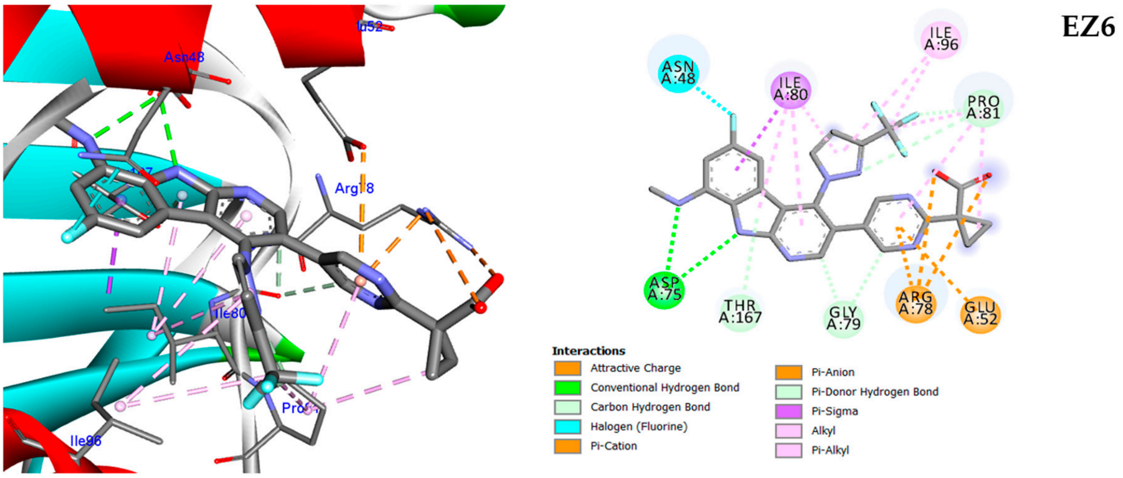

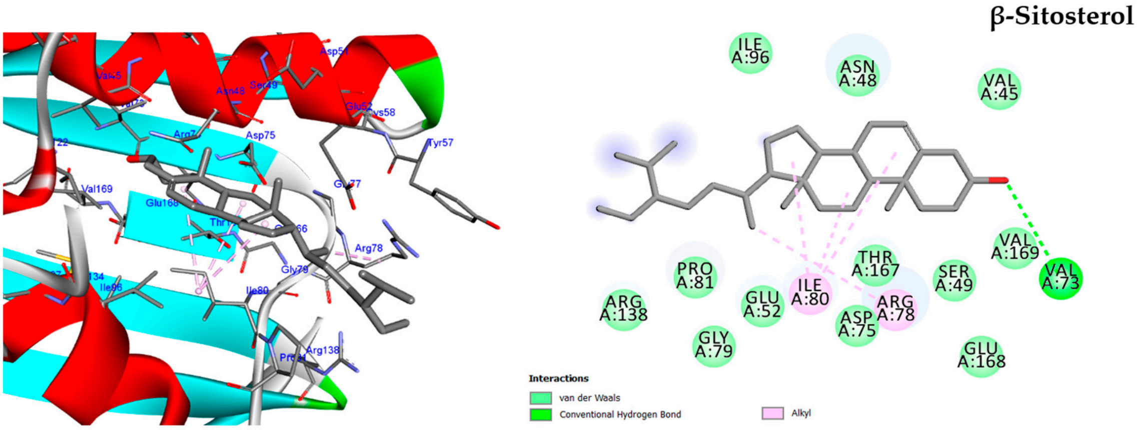

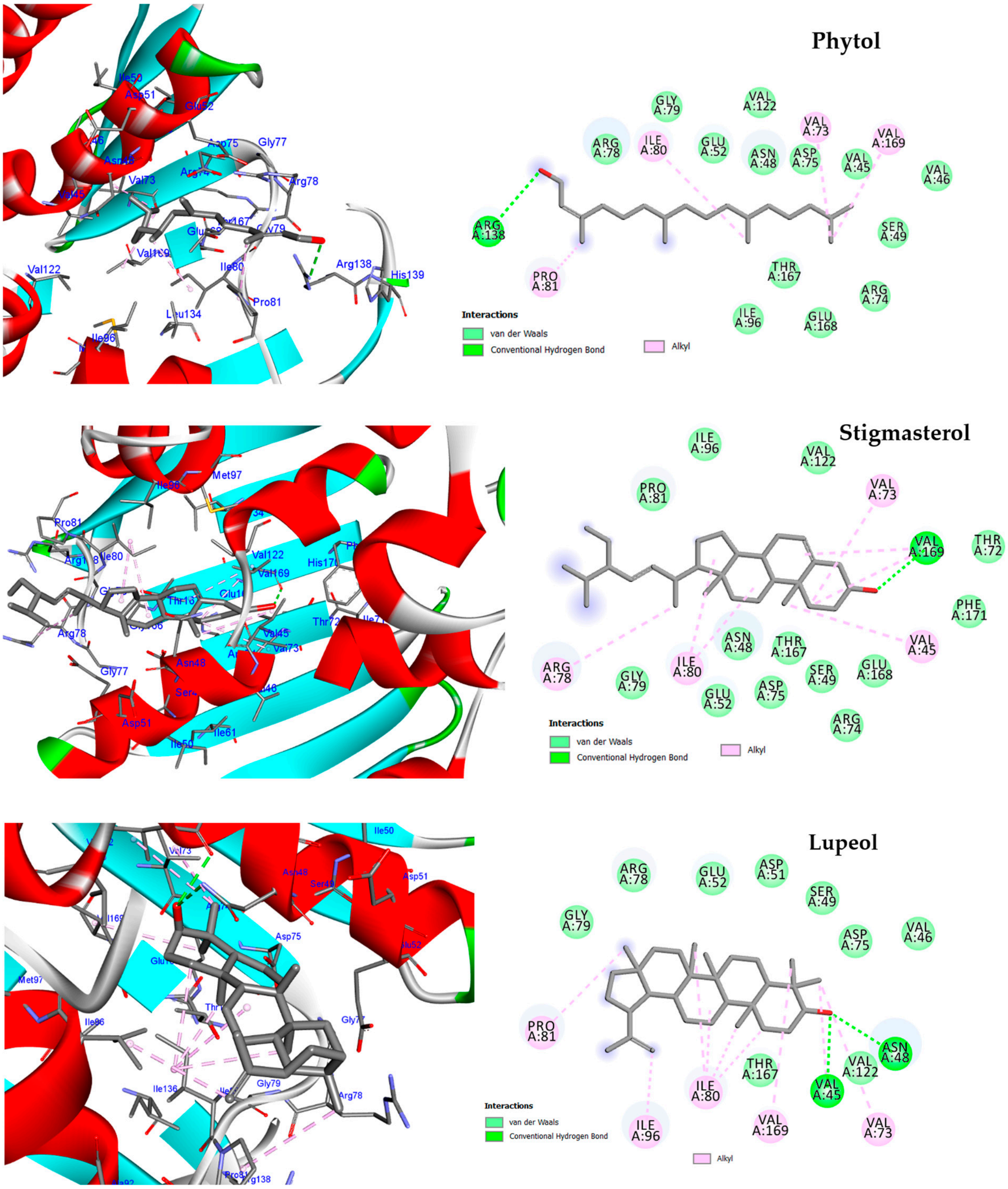

Figure S1: Two-dimensional and three-dimensional images of β-sitosterol, phytol, stigmasterol, lupeol, and EZ6 docked into the active sites of DNA gyrase B enzyme.

Table S4. Types of interactions and docking energy scores of β-sitosterol, phytol, stigmasterol, lupeol, and ceftobiprole against PBP2A.

Penicillin-binding protein (PBP2A), PDB ID: 4dki					
**Compound**	β-Sitosterol	Phytol	Stigmasterol	Lupeol	Ceftobiprole
**Docking energy score (Kcal/ mol)**	-14.45	-12.03	-15.65	-14.20	-15.20
**Amino acids/ Bond type/ Distance (Å)**	Thr444/H-Bond/3.01<br>Tyr519/H-Bond/2.99<br>Met641/Alkyl/3.83<br>Tyr446/Pi-Alkyl/5.12<br>Tyr446/Pi-Alkyl/4.32<br>His583/Pi-Alkyl/4.62	Ser598/H-Bond/2.92<br>His583/H-Bond/2.94<br>Tyr446/Pi-Sigma/3.90<br>Ala601/Alkyl/3.95<br>Tyr446/Pi-Alkyl/5.14	Thr444/H-Bond/3.05<br>Tyr519/H-Bond/3.19<br>Tyr446/Pi-Sigma/3.90<br>Met641/Alkyl/4.71<br>Met641/Alkyl/4.19<br>Tyr446/Pi-Alkyl/4.92<br>Tyr446/Pi-Alkyl/4.71	Gln521/H-Bond/2.75<br>Tyr446/Pi-Sigma/3.57<br>Met641/Alkyl/4.35<br>Ala642/Alkyl/4.13<br>Met641/Alkyl/4.85<br>Met641/Alkyl/3.46<br>Tyr446/ Pi-Alkyl/4.27<br>Tyr446/Pi-Alkyl/5.26<br>Tyr446/Pi-Alkyl/4.72	Lys406/ Attractive Charge/4.93<br>Lys597Attractive Charge/4.13<br>Ser462/H-Bond/3.28<br>Asn464/H-Bond/2.87<br>Gly520/H-Bond/2.84<br>Ser403/H-Bond/3.31<br>Thr600/H-Bond/3.37<br>Ser643/H-Bond/3.03<br>Thr600/Sulfur/3.30<br>Ala601/Pi-Sigma/3.86<br>Trp616/Pi-Sulfur/4.85

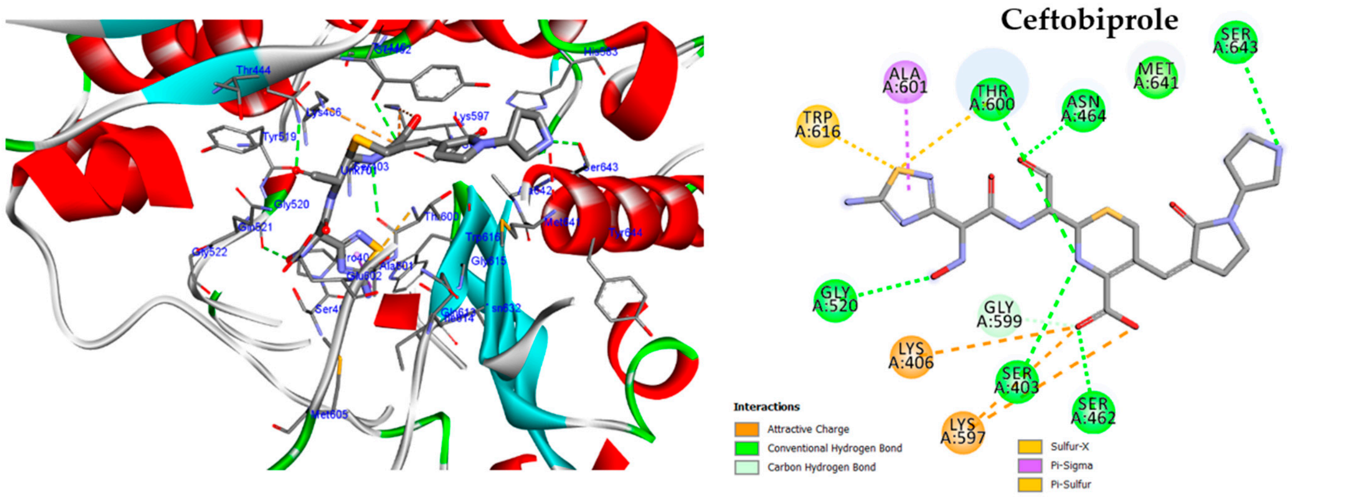

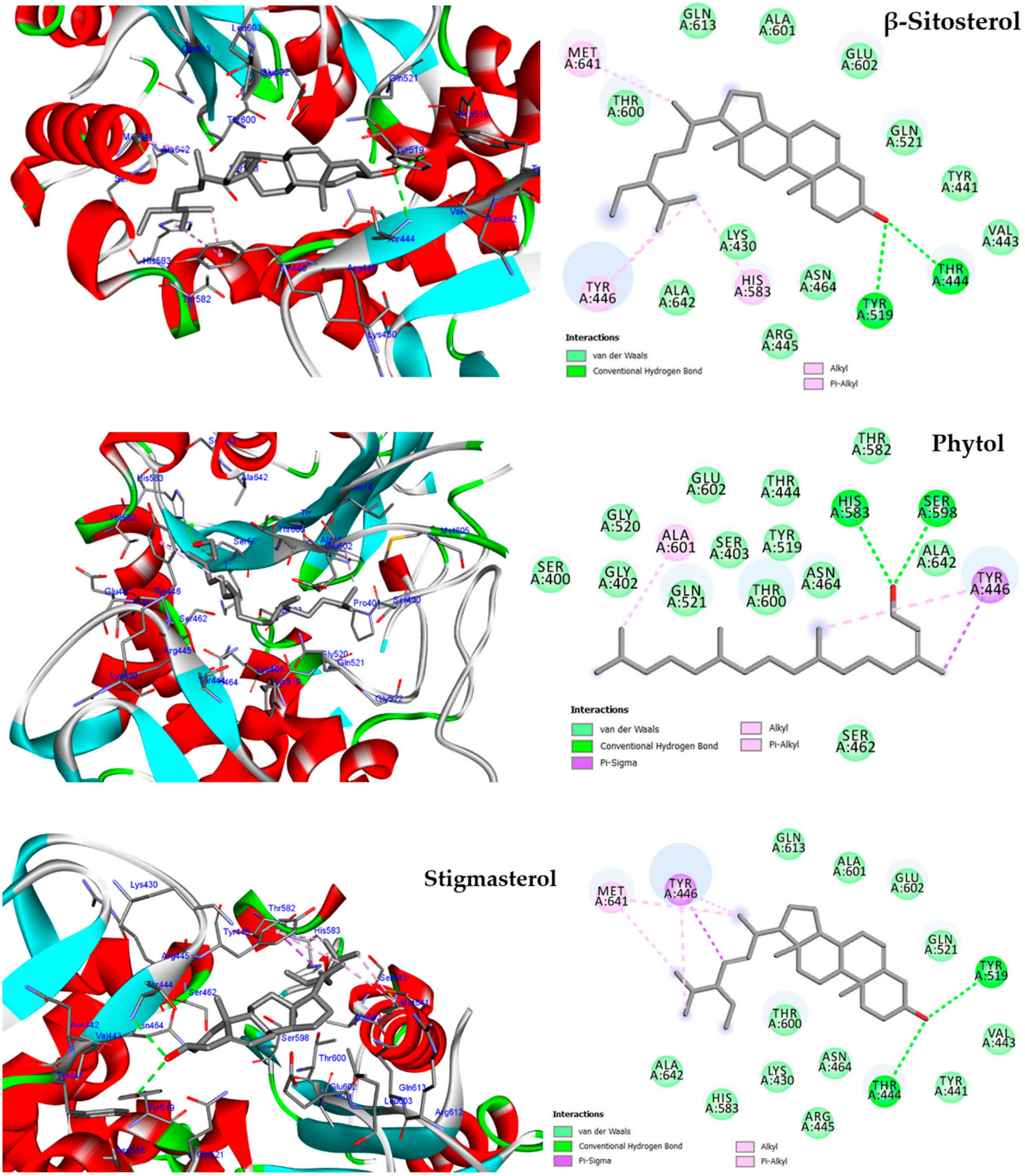
β-Sitosterol
GLN A:613
ALA A:601
MET A:641
GLU A:602
THR A:600
GLN A:521
TYR A:441
VAL A:443
LYS A:430
TYR A:446
ALA A:642
HIS A:583
ASN A:464
THR A:444
TYR A:519
ARG A:445
Interactions
van der Waals
Conventional Hydrogen Bond
Alkyl
Pi-Alkyl
Phytol
THR A:582
GLU A:602
THR A:444
HIS A:583
SER A:598
GLY A:520
ALA A:601
SER A:403
TYR A:519
ALA A:642
SER A:400
GLY A:402
GLN A:521
THR A:600
ASN A:464
TYR A:446
Interactions
van der Waals
Conventional Hydrogen Bond
Pi-Sigma
Alkyl
Pi-Alkyl
SER A:462
Stigmasterol
GLN A:613
ALA A:601
GLU A:602
MET A:641
TYR A:446
GLN A:521
TYR A:519
THR A:600
VAL A:443
ALA A:642
LYS A:430
ASN A:464
THR A:444
TYR A:441
HIS A:583
ARG A:445
Interactions
van der Waals
Conventional Hydrogen Bond
Pi-Sigma
Alkyl
Pi-Alkyl

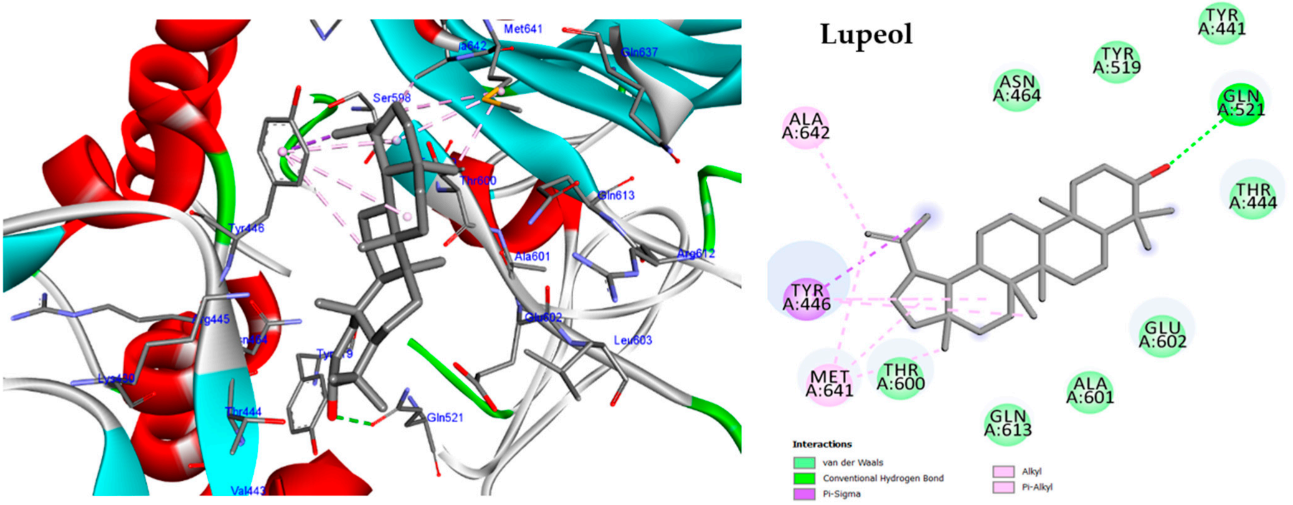

Figure S2: Two-dimensional and three-dimensional images of β-sitosterol, phytol, stigmasterol, lupeol, and ceftobiprole docked into the active sites of PBP2A enzyme.

Table S5. Types of interactions and docking energy scores of β-sitosterol, phytol, stigmasterol, lupeol, and B7O against Influenza A virus nucleoprotein (NP).

**Influenza A virus nucleoprotein, PDB ID: 6j1u**					
**Compound**	β-Sitosterol	Phytol	Stigmasterol	Lupeol	Co- crystal Ligand (B7O)
**Docking energy score (Kcal/ mol)**	-8.81	-8.38	-8.32	-9.08	-6.31
**Amino acids/ Bond type/ Distance (Å)**	Ser283/H-Bond/2.88<br>Cys279/H-Bond/3.17<br>Ile41/Alkyl/4.69<br>Cys44/Alkyl/4.02<br>Cys44/Alkyl/3.36<br>Lys48/Alkyl/4.485	Arg55/H-Bond/2.95<br>Asp51/H-Bond/2.97<br>Asp51/H-Bond/3.06<br>Ser283/C-H-Bond/3.77<br>Arg55/Alkyl/4.56<br>Cys44/Alkyl/4.89<br>Try40/Pi-Alkyl/5.40	Ser283/H-Bond/2.84<br>Ile41/Alkyl/5.30<br>Cys44/Alkyl/4.11<br>Ala286/Alkyl/5.27<br>Ala286/Alkyl/3.66<br>Ile41/Alkyl/3.86<br>Cys44/Alkyl/3.85<br>Ile41/Alkyl/5.24	Ser283/H-Bond/2.85<br>Ser283/H-Bond/3.38<br>Cys279/H-Bond/3.32<br>Cys44/Alkyl/4.10<br>Ala286/Alkyl/3.19<br>Ile41/Alkyl/4.20<br>Cys44/Alkyl/5.18<br>Arg55/Alkyl/4.13<br>Ile41/Alkyl/4.34<br>Cys44/Alkyl/3.45	Gly54/C-H-Bond/3.53<br>Asp51/C-H-Bond/2.98<br>Ser283/ C-H-Bond/2.84<br>Asp51/Pi-Anion/4.35<br>Cys44/Pi-Sulfur4.35<br>Ile41/Pi-Alkyl/5.41<br>Ile41/Pi-Alkyl/4.94<br>Ala286/Pi-Alkyl/4.67

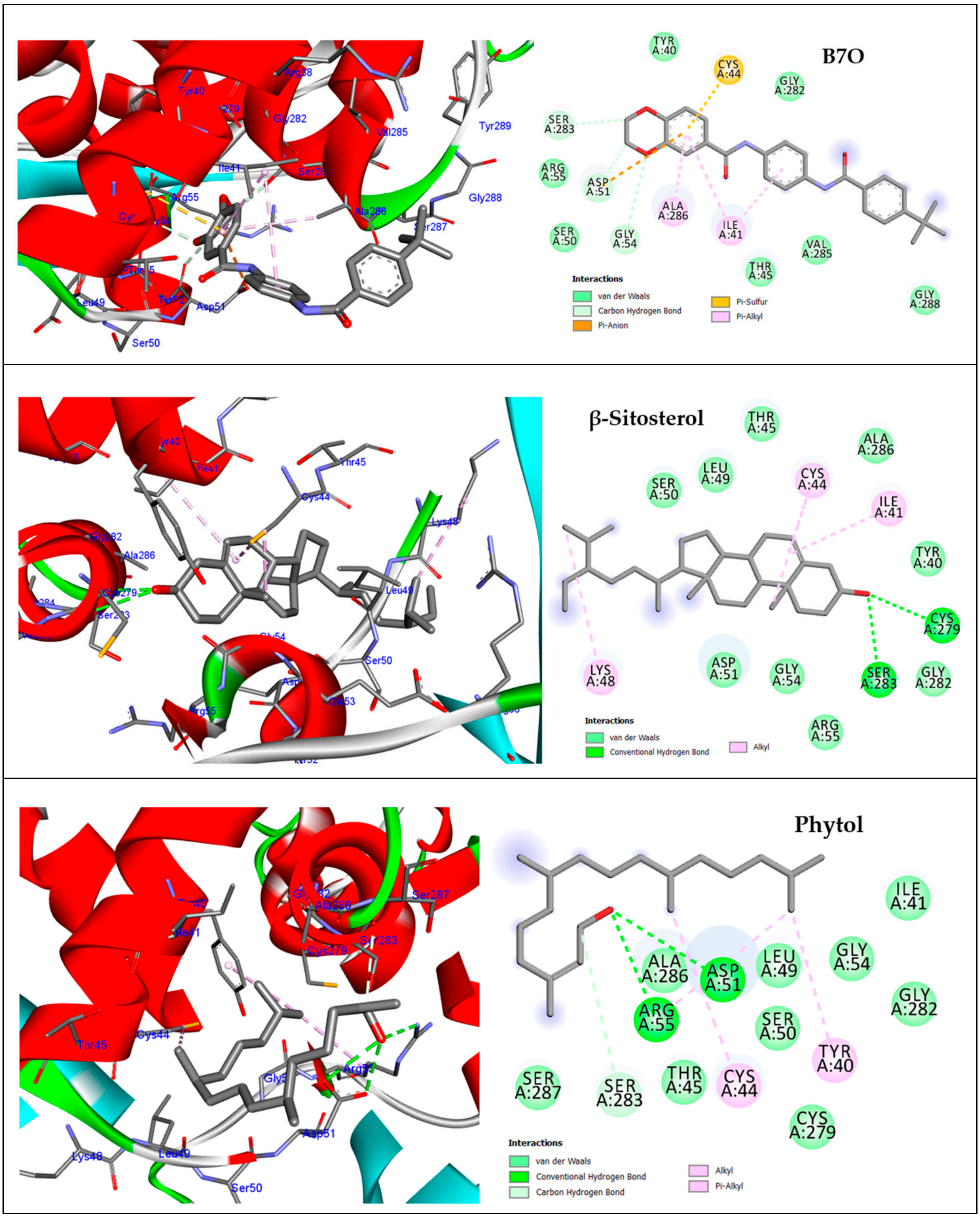
B7O
TYR A:40
CYS A:44
GLY A:282
SER A:283
ARG A:55
ASP A:51
ALA A:286
ILE A:41
SER A:50
GLY A:54
VAL A:285
THR A:45
GLY A:288
Interactions
van der Waals
Carbon Hydrogen Bond
Pi-Anion
Pi-Sulfur
Pi-Alkyl
β-Sitosterol
THR A:45
ALA A:286
SER A:50
LEU A:49
CYS A:44
ILE A:41
TYR A:40
CYS A:279
LYS A:48
ASP A:51
GLY A:54
SER A:283
GLY A:282
ARG A:55
Interactions
van der Waals
Conventional Hydrogen Bond
Alkyl
Phytol
ILE A:41
ALA A:286
ASP A:51
LEU A:49
GLY A:54
GLY A:282
ARG A:55
SER A:50
TYR A:40
SER A:287
SER A:283
THR A:45
CYS A:44
CYS A:279
Interactions
van der Waals
Conventional Hydrogen Bond
Carbon Hydrogen Bond
Alkyl
Pi-Alkyl

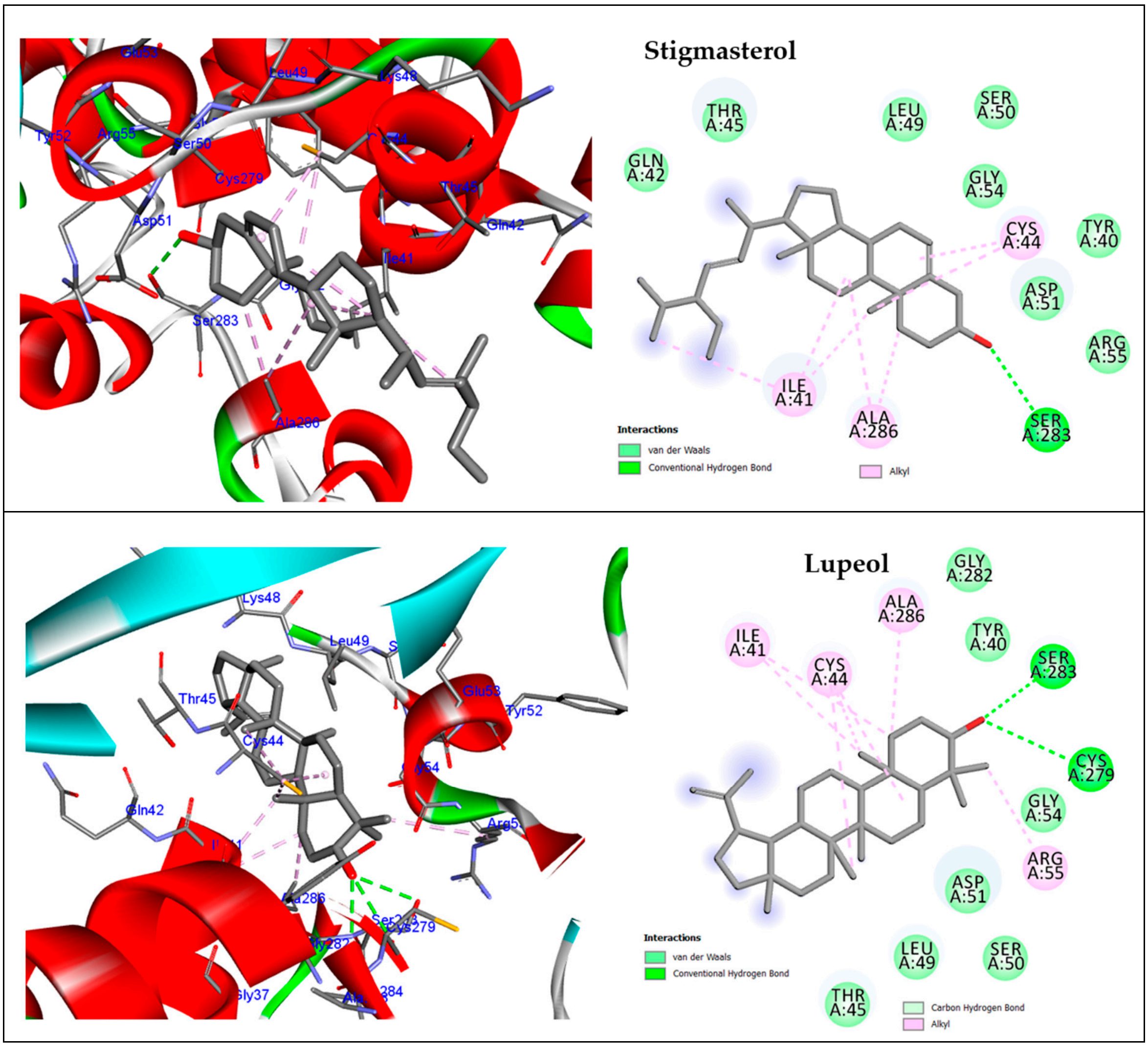

Figure S3: Two-dimensional and three-dimensional images of β-sitosterol, phytol, stigmasterol, lupeol, and B7O docked into the active sites of Influenza A virus nucleoprotein (NP).

Table S6. Types of interactions and docking energy scores of β-sitosterol, phytol, stigmasterol, lupeol, and 21G against Influenza A virus polymerase.

**Influenza A virus polymerase, PDB ID: 4p1u**					
**Compound**	β-Sitosterol	Phytol	Stigmasterol	Lupeol	Co- crystal Ligand (21G)
**Docking energy score (Kcal/ mol)**	-9.68	-10.68	-11.04	-8.38	-8.07

**Amino acids/ Bond type/ Distance (Å)**	Phe404/H-Bond/3.23<br>Phe404/Pi-Donor-H-Bond/3.37<br>Met426/Alkyl/4.39<br>Phe323/Pi-Alkyl/4.04<br>Phe325/ Pi-Alkyl/4.82<br>His357/Pi-Alkyl/3.83<br>His357/Pi-Alkyl/4.19<br>Phe404/Pi-Alkyl/4.95<br>Phe404/Pi-Alkyl/5.27<br>His427/Pi-Alkyl/5.35	Arg332/H-Bond/2.98<br>Ser337/H-Bond/3.04<br>Phe323/Pi-Sigma/3.55<br>Met426/Alkyl/4.13<br>Lys376/Alkyl/5.23<br>Phe323/Pi-Alkyl/5.47<br>His357/Pi-Alkyl/4.52<br>Phe363/Pi-Alkyl/4.69<br>Phe404/Pi-Alkyl/4.01	Arg332/H-Bond/2.03<br>Arg332/H-Bond/2.89<br>Ser337/H-Bond/2.20<br>Phe323/Pi-Alkyl/5.24<br>Phe323/Pi-Alkyl/5.41<br>Phe323/Pi-Alkyl/4.38<br>Phe323/Pi-Alkyl/5.32<br>Phe323/Pi-Alkyl/5.27<br>His357/Pi-Alkyl/4.93<br>His357/Pi-Alkyl/5.24<br>His357/Pi-Alkyl/4.49<br>His357/Pi-Alkyl/5.28<br>His427/Pi-Alkyl/4.49<br>His427/Pi-Alkyl/4.92	Ser321/C-H-Bond/2.63<br>Met426/Alkyl/5.29<br>Arg355/Alkyl/4.74<br>Phe323/Pi-Alkyl/4.83<br>Phe323/Pi-Alkyl/3.34<br>Phe323/Pi-Alkyl/5.15<br>His357/Pi-Alkyl/5.28<br>His357/Pi-Alkyl/5.16<br>His357/Pi-Alkyl/4.80	Lys339/Salt bridge/ 3.21<br>Lys339/Attractive charge/4.21<br>Arg355/Attractive charge/3.37<br>Arg355/H-Bond/2.90<br>Lys376/H-Bond/3.13<br>Gln406/H-Bond/3.21<br>Glu361/H-Bond/2.83<br>Phe404/C-H-Bond/ 3.34995<br>Phe404/Halogen/3.43<br>Phe323/ Pi-Pi Stacked/3.67<br>His357/ Pi-Pi Stacked/3.75<br>His357/ Pi-Pi Stacked/3.49<br>His357/ Pi-Pi Stacked/5.10<br>Phe404/ Pi-Pi Stacked/3.86<br>Phe323/ Pi-Pi Stacked/5.36<br>Phe404/ Pi-Pi Stacked/4.87<br>Met426/Alkyl/4.65<br>His427/Pi-Alkyl/5.18

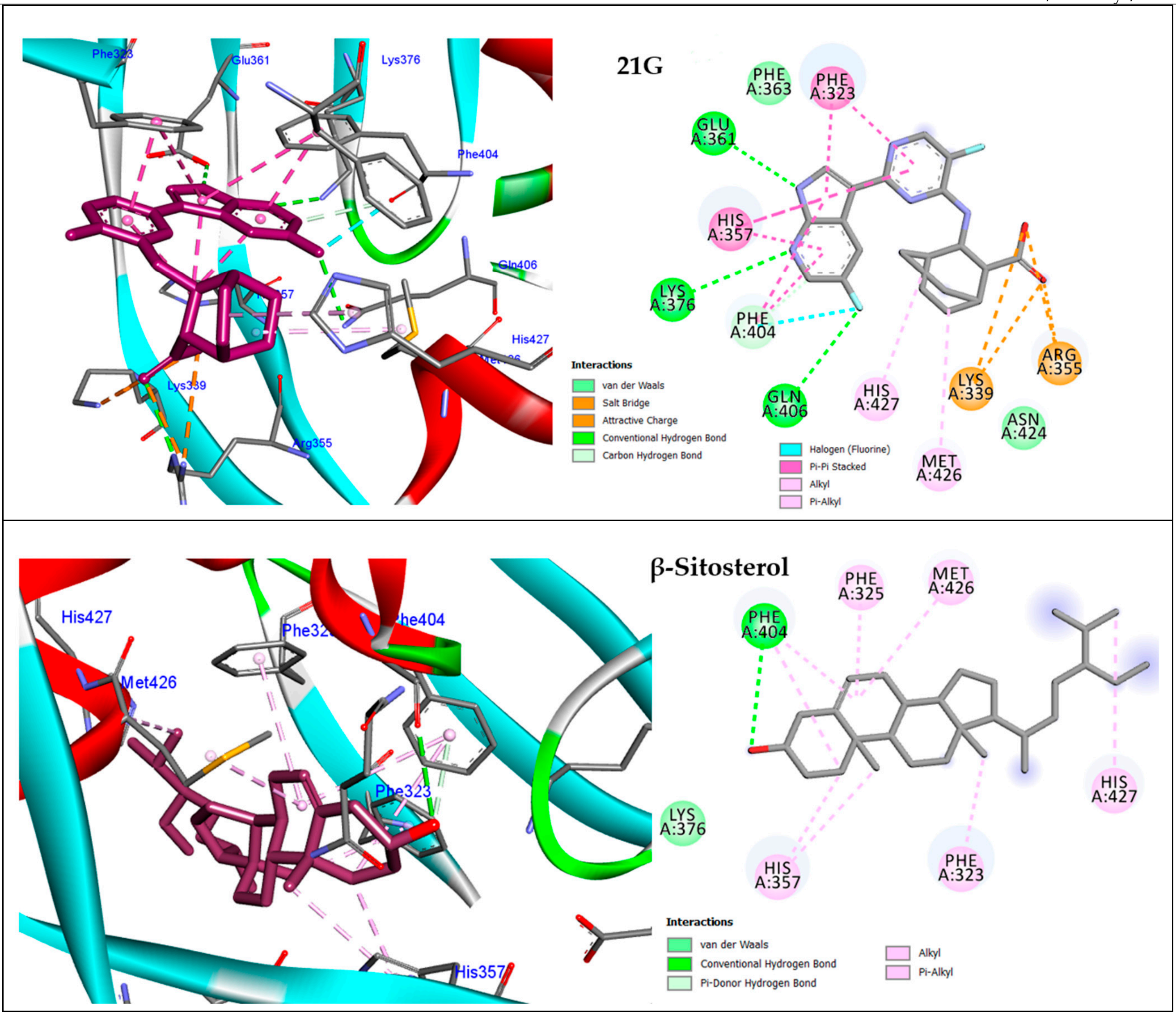

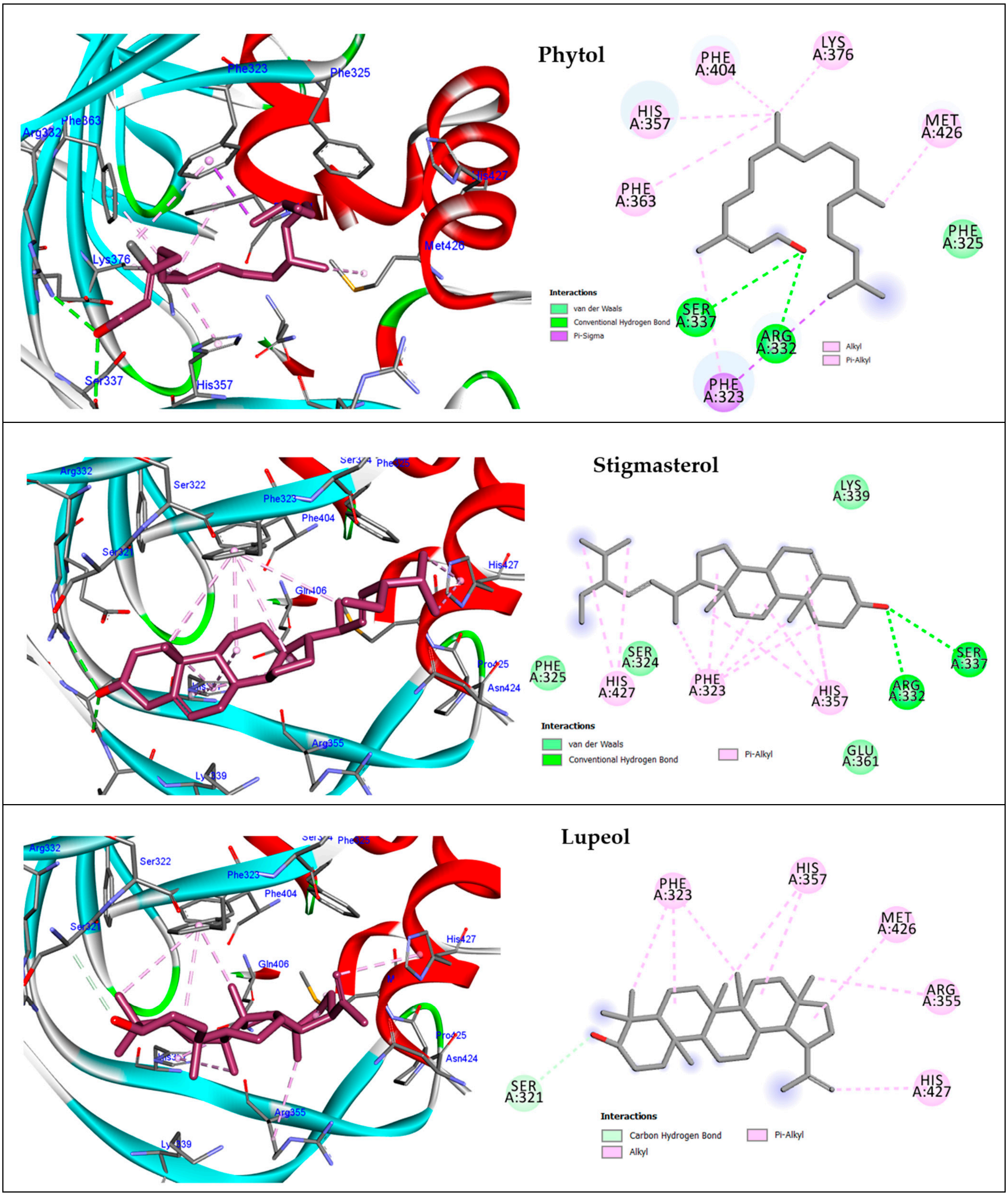

Figure S4: Two-dimensional and three-dimensional images of β-sitosterol, phytol, stigmasterol, lupeol, and 21G docked into the active sites of Influenza A virus polymerase.
